# Study of 2-aminoquinolin-4(1H)-one under Mannich and retro-Mannich reaction

**DOI:** 10.1371/journal.pone.0175364

**Published:** 2017-05-30

**Authors:** Petr Funk, Kamil Motyka, Miroslav Soural, Michal Malon, Hiroyuki Koshino, Joachim Kusz, Jan Hlavac

**Affiliations:** 1 Department of Organic Chemistry, Institute of Molecular and Translational Medicine, Faculty of Science, Palacky University, Olomouc, Czech Republic; 2 Institute of Molecular and Translational Medicine, Faculty of Medicine and Dentistry, Palacký University, Olomouc, Czech Republic; 3 JEOL RESONANCE Inc., Akishima, Tokyo, Japan; 4 RIKEN Center for Sustainable Resource Science, Wako, Saitama, Japan; 5 Institute of Physics, University of Silesia, Poland; Bangor University, UNITED KINGDOM

## Abstract

2-Aminoquinolin-4(1H)-one was reacted with various primary/secondary amines and paraformaldehyde under Mannich reaction conditions. In the case of secondary amines, the reaction in N,N-dimethylformamide yielded expected Mannich products accompanied with 3,3'-methylenebis(2-aminoquinolin-4(1H)-one). Except these main products, the pyrimido[4,5-b]quinolin-5-one derivative was also identified as co-product. The reaction with primary amines led to the formation of pyrimido[4,5-b]quinolin-5-ones. The Mannich reaction products were thermally unstable and afforded a mixture of bis-(2-aminoquinolin-4(1H)-one) and tris-(2-aminoquinolin-4(1H)-one) derivative, probably via reactive methylene species. This retro-Mannich reaction was tested in reaction with indole and thiophenole as nucleophilles, and appropriate conjugates were formed. The mechanism of above discussed reactions in which 2-aminoquinolinone displays the nucleophilicity on C3 carbon as well as N2 nitrogen is discussed.

## Introduction

Due to the outstanding position of the quinolin-4(1*H*)-one scaffold in the field of medicinal chemistry, 2-aminoquinolin-4(1*H*)-ones have been widely studied as potential pharmacological agents in different areas. The first paper in this field published in 1974 was devoted to the synthesis and evaluation of antimicrobial activity of selected 2-amino-4-alkoxyquinolines. [1] Recently, 3-acetyl-2-aminoquinolin-4(1*H*)-ones were reported as potent and selective calpain inhibitors. [2] 2-[2-Substituted-3-(3,4-dichlorobenzylamino)propylamino]qui-nolin-4-ones were found to possess antibacterial activity against various strains, mainly Staphylococcus aureus and Enterococci.[3] Derivatives of 2-aminoquinolin-4-ol have been identified as suitable structural motifs for the preparation of novel oligonucleotide conjugates to enhance binding affinities for complementary RNA targets.[4]

Furthermore, the latest results show that compounds based on 2-aminoquinolin-4-ol promote a significant telomere dysfunction leading to long-term anti-tumor activity.[5–8] The same scaffold was included in the structure of ferrocenes with leishmanicidal activity.[9] Although the number of 2-aminoquinolin-4(1*H*)-one derivatives were described, they were almost exclusively synthesized by scaffold construction. Modification of 2-amino-4-alkoxyquinoline scaffold was described rarely. The attack of the C^3^ carbon with electrophiles was reported only for a coupling reaction with aryl diazonium salts yielding corresponding azocompounds. [10] Formation of benzo[b][1,8]naphthyridine scaffold was described via a reaction with 3-formylchromone [11] or arylmalonates,[12,13] followed by the condensation of the amino group in the position 2. The reaction of amino group itself was reported only in an acylation reaction.[10,14]

The combination of the nucleophilic C^3^ carbon and the amino group in position 2 is challenging for the potential use of 2-aminoquinolin-4(1*H*)-one as the starting material in the Mannich reaction, in which the compound can behave as both C- and N-nucleophile. Although the Mannich reaction belongs to one of the most powerful synthetic strategies for carbon-carbon bond formation and has found numerous applications in the syntheses of natural and biologically active compounds,[15,16] little attention was given to its use for the modification of quinolin-4(1*H*)-ones. Only several studies were reported, in which the Mannich reaction was used for the modification of 2-methyl-quinolin-4(1*H*)-ones [17,18] with the aim to prepare novel antibacterial agents.

In this article, we report the results of the study of 2-aminoquinolin-4(1H)-one modification via the Mannich reaction to enlarge the portfolio of synthetic strategies applicable for the preparation of new biologically relevant compounds.

## Results and discussion

### Synthesis

**Fig 1:**
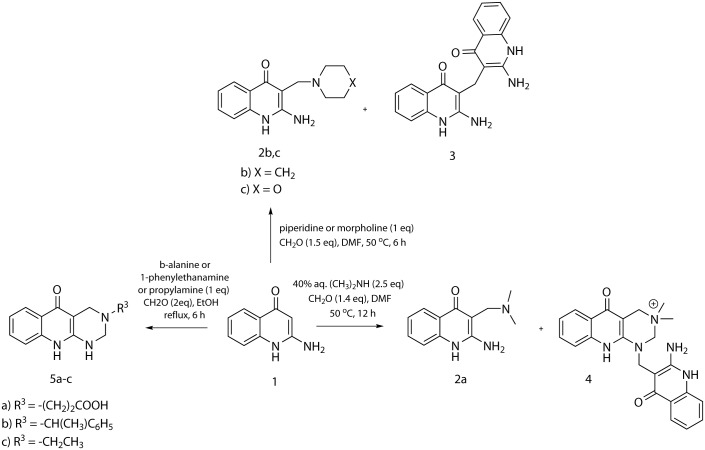
Reaction of 2-aminoquinolin-4(1H)-one with primary and secondary amines.

The study of the Mannich reaction employing 2-aminoquinolin-4(1H)-one **1** was performed with use of selected primary amines (β-alanine, 1-phenylethanamine, propylamine) and secondary amines (dimethylamine, piperidine, morpholine) (Scheme 1). Although the Mannich reaction of aminoquinolinone **1** with secondary amines afforded mainly the expected compounds **2a-c**, it was also accompanied with numerous by-products. In the case of morpholine and piperidine, the major by-product in a yield ranging from 15 to 20% was isolated and identified as 3,3’-methylenebis(2-aminoquinolin-4(1*H*)-one) **3**. In the case of dimethylamine, the expected product **2a** was accompanied with pyrimido[4,5-b]quinolin-5-one **4** formed in a yield of 25%. When reaction was carried out in ethanol instead of N,N-dimethylformamide (DMF), pure compounds **2a-c** without the formation of side products were isolated. In contrast to secondary amines, the Mannich reaction with primary amines in ethanol did not provide expected products, but formation of tetrahydropyrimidine derivatives **5a-c** was observed. As it was expected, the purity and yield of compounds **5a-c** were higher when the quantity of paraformaldehyde was raised to 2 equiv. (Fig 1).

**Fig 2:**
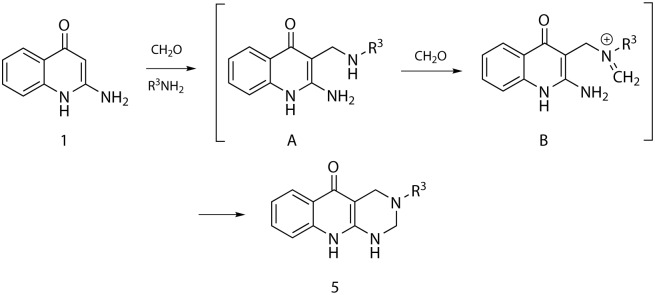
Plausible mechanism of the reaction yielding tetrahydropyrimido[4,5-b]quinolin-5-ones 5.

Formation of tetrahydropyrimido[4,5-b]quinolin-5-ones **5** clearly demonstrates the ability of 2-amino-4(1*H*)-quinolinone to act as both C/N-nucleophile in the cascade reaction. The reaction mechanism probably involved formation of the standard Mannich-type intermediate **A**, which was converted by paraformaldehyde to the corresponding iminium salt **B**. The reaction sequence was accomplished by the intramolecular nucleophilic addition to give the tetrahydropyrimidine scaffold of derivative **5** (Fig 2).

**Fig 3:**
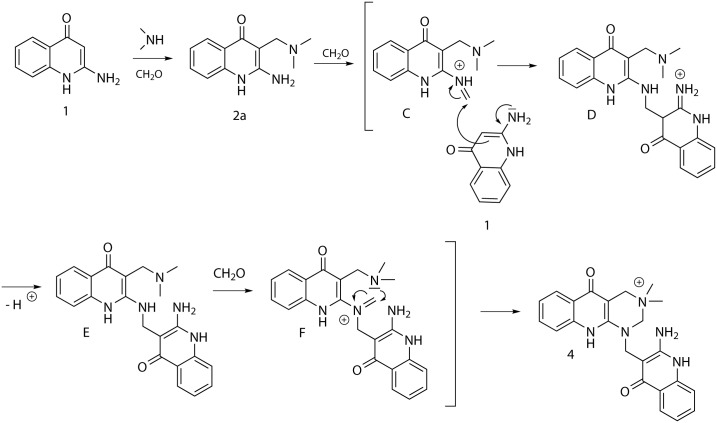
Plausible mechanism of the reaction yielding pyrimido[4,5-b]quinolin-3-ium salt 4.

A similar reaction was undoubtedly responsible for the formation of compound **4** (Scheme 3) from the starting material **1**, in situ formed derivative **2a** and paraformaldehyde. A significant role was probably played by different nucleophilicity of amino groups in intermediate **E**, in which the secondary amine reacts predominantly with formaldehyde to afford iminium salt **F**, which is subsequently transformed to final product **4**. (Fig 3)

**Fig 4:**
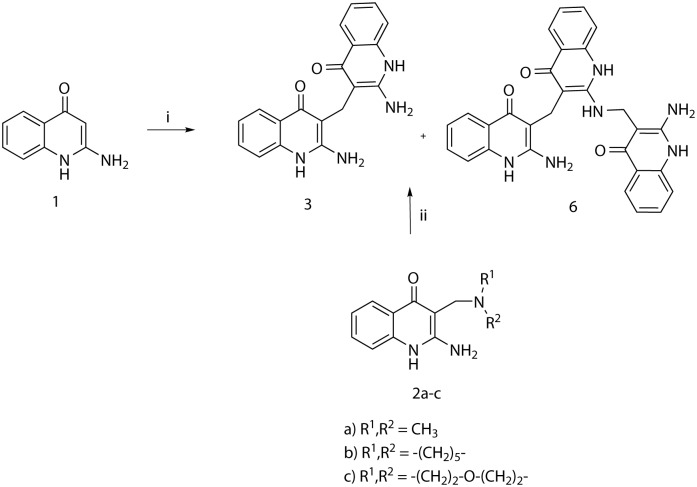
Transformation of 2-aminoquinolin-4(1H)-one and Mannich products 2 to bis and tris-quinolinone derivatives.

When the aminoquinolinone **1** was treated only with paraformaldehyde, the quinolinone dimer **3** was formed as the main product at ambient temperature, while at 90°C a significant amount of derivative **6** was observed as co-product. More surprisingly, the same mixture of products was observed in LC/MS spectra when Mannich derivatives **2a-c** were heated in DMF at the same temperature (Fig 4).

**Fig 5:**
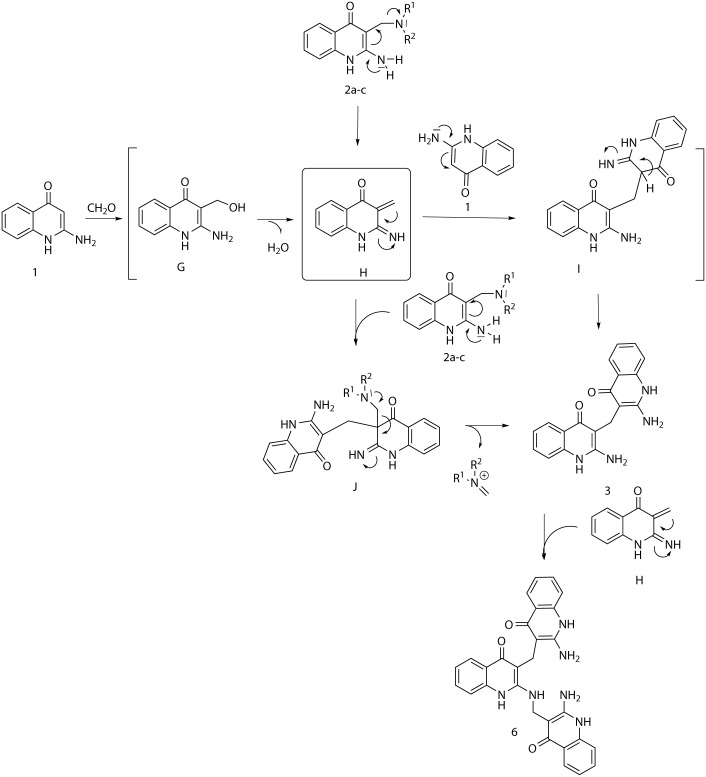
Plausible mechanism leading to derivatives 3 and 6.

This fact can be explained by the possible formation of the intermediate **H** originating from the reaction of quinolinone **1** with paraformaldehyde or from decomposition of Mannich products **2a-c** (Fig 5). The intermediate **H** reacts under Michael addition with 2-aminoquinolin-4(1H)-one **1** to afford intermediate **I**, followed by the final tautomerization yielding the product **3** (Fig 5). The dimerization of the similar 2-amino-quinolinone derivatives via reaction of quinolinone with paraformaldehyde was previously observed by Bany et al.,[19] but the mechanism has not been discussed to date. Tris-(2-aminoquinolin-4(1H)-one) **6** was finally formed by reaction of derivative **3** with in-situ generated intermediate **H** (Fig 5).

Our effort to prove the existence of intermediate **G** or **H** was not successful, probably due to their instability and rapid transformation to the product **3**. When 2-aminoquinolin-4(1*H*)-one **1** was subjected to the reaction with paraformaldehyde (1, 3 or 6 equiv.) at ambient temperature without the presence of amines, only the target 3,3’-methylenebis(2-aminoquinolin-4(1H)-one) **3** was obtained, whereas the suggested intermediates **G** or **H** were not detected.

**Fig 6:**
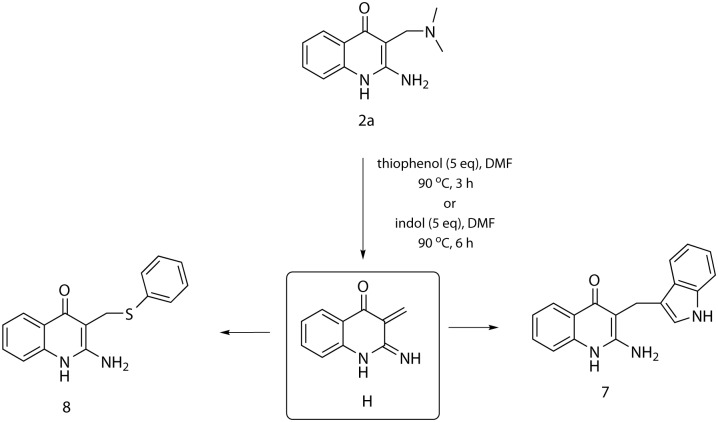
Reaction of Mannich product 2a with indole and thiophenol.

The theory of the intermediate **H** formation was indirectly confirmed by the method of crossed reactions when compound **2a** was heated in the presence of indol as a concurrent C-nucleophile. In accordance with our expectation, the corresponding 3-((1H-indol-3-yl)methyl)-2-aminoquinolin-4(1H)-one **7** was isolated. This fact points to the retro-Mannich mechanism of the reaction (Fig 6). When thiophenol was used instead of indol, the sulphidic derivative **8** was obtained.

### Structural analysis of prepared compounds

**Fig 7:**
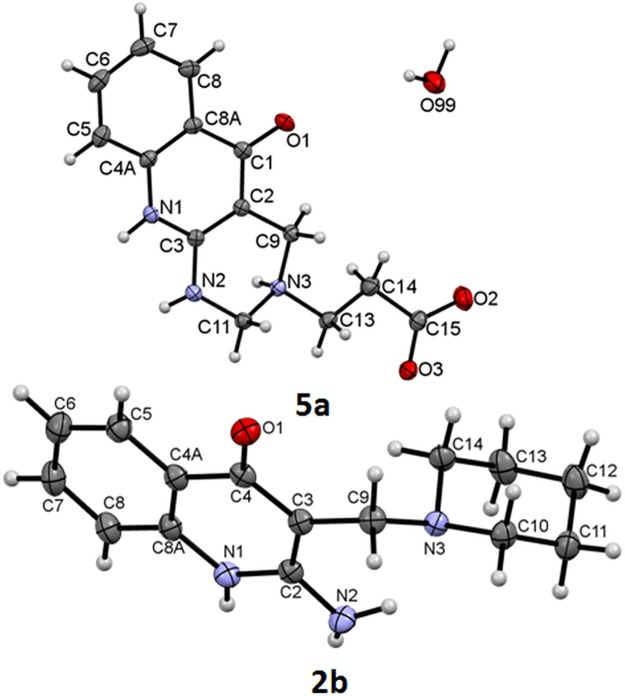
ORTEP view of compounds 2b and 5a. Displacement ellipsoids are drawn at the 50% probability level.

Molecular structures of all compounds were determined by solution NMR spectroscopy. For the univocal structure determination of compound **6** measurements and elaborate analysis of 1D and 2D spectra, including 1H-15N correlation spectra and spectra recorded at variable temperature were performed. The spectra and their detailed discussion are given in Supplementary Information (see S20, S21, S34 and S37–S43 Figs). In addition to NMR spectroscopy, structures of derivatives **2b** and **5a** were unambiguously confirmed by single-crystal X-ray analysis (Fig 7).

## Conclusion

In this article, we reported the study of 2-amino-4(1H)-quinolinone reactivity under Mannich reaction conditions. Apart from expected products, the reaction provided various unexpected compounds exhibiting interesting structures. Further, we observed a thermal instability of Mannich products leading to the plausible formation of reactive methylene intermediate, which can allow synthesis of polycyclic heterocycles via retro-Mannich reaction or enable conjugation with other nucleophiles. The developed procedures can be applied not only for modification of 2-amino-4(1H)-quinolinone, but also for a synthesis of quite new heterocyclic scaffolds with application in any area of chemistry.

## Materials and methods

### Apparatus

Solvents and chemicals were purchased from Aldrich (Milwaukee, IL, http://www.sigmaaldrich.com), Acros (Geel, Belgium, http://www.acros.cz) and Fisher (Pittsburgh, PA, http://www.fishersci.com).

LCMS analyses were measured with Thermo Exactive plus instrument (Thermo Scientific, USA). The chromatographic apparatus consisted of Dionex Ultimate 3000 LC pump, autosampler and column thermostat. The separation was performed on a Gemini C18, 3 μm, 50x2 mm i.d. column (Phenomenex, USA) using isocratic elution. The mobile phase comprised acetonitrile/ water 80/20 + 0.1% of formic acid. The flow rate was kept at 300 μL/min, the column temperature was 25°C. Sample preparation: 1 mg/1 mL acetonitrile+dil. 5 μL/1 mL acetonitrile/water 8/2 before injection of 3 μL.

High resolution mass spectrometer Exactive based on orbitrap mass analyser was equipped with Heated Electrospray Ionization (HESI). The spectrometer was tuned to obtain maximum response for m/z 70–800. The source parameters were set to the following values: HESI temperature 150°C, spray voltage +3.5 kV, -3 kV; transfer capillary temperature 320°C, sheath gas/aux gas (nitrogen) flow rates 40/20. The HRMS spectra of target peaks allowed to evaluate their elemental composition with less than 1 ppm difference between experimental and theoretically calculated value.

^1^H/^13^C/^15^N NMR spectra were obtained on Bruker (300 MHz), Varian (400 MHz), JEOL ECA400II (400 MHz), JEOL ECZ500R (495 MHz) and JEOL ECA600 (600 MHz) instruments. NMR spectra were recorded at temperature from -50 to +25°C in DMSO-d6 or DMF-d7 solutions and referenced to the residual signal of DMSO-d6 or DMF-d7 (for 1H NMR: DMSO-d6 (2.50), DMF-d7 (2.75); for ^13^C NMR: DMSO-d6 (39.51), DMF-d7 (29.76); for ^15^N NMR: DMF-d7 (103.2)). Chemical shifts δ are reported in ppm and coupling constants J in Hz.

The compounds were purified by reversed phase semipreparative HPLC chromatograph (Agilent Technologies, 1200 Series, USA) consisted of two pumps enabling high-pressure gradient elution, manual valve injector with 1-ml injection loop, UV-VIS detector and fraction collector. The column C18 Pro (particle size 5 μm, length 100mm, I. D. 20 mm, YMC, Japan) was applied for chromatographic separation. The linear gradient elution consisted of 80:20% 0.01 M ammonium acetate buffer:acetonitrile to 10:90% in 13 min and then the composition of mobile phase was kept for 2 min to wash the column. The column was isocraticaly equilibrated for 5 min for next separations. The mobile phase flow rate was set to 15 mL min-1. 200 μL of crude sample was repeatedly injected for separation. The software ChemStation (version B 04.02) was applied for controlling of the instrument and data evaluation.

### Synthetic procedures

#### General procedure for compounds 2a-c

2-Amino-1H-quinolin-4-one **1** (200 mg, 1.2 mmol) was dissolved in ethanol (5 mL) followed by addition of paraformaldehyde (37.5 mg, 1.2 mmol) and secondary alkylamine* (1.2 mmol). The mixture was stirred at 50°C for 6 hours. The solvent was evaporated in vacuum and the residual solid was suspended in water. The resulting product **2** was filtered off and dried.

* dimethylamine as 40% aqueous solution

**2-Amino-3-((dimethylamino)methyl)quinolin-4(1H)-one (2a)**:

Yield: 165 mg (61%).

^1^H NMR (400 MHz, DMSO-d6) δ ppm 10.75 (br s, 1H), 7.96 (br d, J = 7.8 Hz, 1H), 7.44 (br dd, J = 8.0, 7.0 Hz, 1H), 7.25 (br d, J = 8.0 Hz, 1H), 7.12 (br dd, J = 7.8, 7.0 Hz, 1H), 6.34 (s, 2H), 3.42 (s, 2H), 2.13 (s, 6H).

^13^C NMR (101 MHz, DMSO-d6) δ ppm 173.4, 153.1, 137.8, 130.0, 125.1, 122.7, 121.3, 116.2, 96.3, 52.7, 44.3.

HRMS (ESI): m/z calcd for [C_12_H_15_N_3_O + H]^+^ 218.1288; found 218.1289.

**2-Amino-3-(piperidin-1-ylmethyl)quinolin-4(1H)-one (2b)**:

Yield: 225 mg (70%).

^1^H NMR (400 MHz, DMSO-d6) δ ppm 10.74 (br s, 1H), 7.94 (dd, J = 8.0, 1.3 Hz, 1H), 7.44 (ddd, J = 8.1, 7.1, 1.3 Hz, 1H), 7.24 (br d, J = 8.1 Hz, 1H), 7.12 (ddd, J = 8.0, 7.1, 1.0 Hz, 1H), 6.45 (s, 2H), 3.47 (s, 2H), 2.33 (br, 4H), 1.48 (br, 4H), 1.39 (br, 2H).

^13^C NMR (101 MHz, DMSO-d6) δ ppm 173.6, 153.1, 137.8, 130.0, 125.0, 122.7, 121.3, 116.2, 95.5, 53.3, 52.3, 25.7, 24.2.

HRMS (ESI): m/z calcd for [C_15_H_19_N_3_O + H]^+^ 258.1601; found 258.1602.

**2-Amino-3-(morpholinomethyl)quinolin-4(1H)-one (2c)**:

Yield: 220 mg (68%).

^1^H NMR (300 MHz, DMSO-d6) δ ppm 10.49 (br s, 1 H), 7.96 (dd, J = 8.0, 1.3 Hz, 1H), 7.46 (ddd, J = 8.1, 7.0, 1.3 Hz, 1H), 7.27 (br d, J = 8.1 Hz, 1H), 7.14 (ddd, J = 8.0, 7.0, 0.8 Hz, 1H), 6.44 (s, 2H), 3.66 (s, 2H), 3.60 (br, 4H), 2.54 (br, 4H).

^13^C NMR (75 MHz, DMSO-d6) δ ppm 174.1, 153.2, 137.8, 130.4, 125.0, 122.4, 121.5, 116.1, 93.7, 65.6, 52.2, 51.4.

HRMS (ESI): m/z calcd for [C_14_H_17_N_3_O_2_ + H]^+^ 260.1394; found 260.1394.

**Preparation of 3**,**3'-methylenebis(2-aminoquinolin-4(1H)-one) (3)**

2-Amino-1H-quinolin-4-one **1** (100 mg, 0.6 mmol) was dissolved in DMF (3 mL) followed by addition of paraformaldehyde (9.4 mg, 0.3 mmol). The mixture was stirred at 90°C for 5 hours and then cooled to room temperature. The precipitate product was filtered off, washed with water and dried. Yield: 97 mg (93%).

^1^H NMR (400 MHz, DMSO-d6) δ ppm 10.92 (s, 2H), 8.00 (dd, J = 8.0, 1.2 Hz, 2H), 7.50 (br s, 4H), 7.45 (ddd, J = 8.1, 7.1, 1.2 Hz, 2H), 7.27 (br d, J = 8.1 Hz, 2 H), 7.15 (ddd, J = 8.0, 7.1, 0.7 Hz, 2H), 3.67 (s, 2H).

^13^C NMR (101 MHz, DMSO-d6) δ ppm 173.7, 153.6, 137.2, 130.0, 124.7, 122.0, 121.4, 116.0, 101.1, 17.8.

HRMS (ESI): m/z calcd for [C_19_H_16_N_4_O_2_ + H]^+^ 333.1346; found m/z 333.1344.

**Preparation of 1-((2-aminoquinolin-4(1H)-one-3-yl)methyl)-3**,**3-dimethyl-5-oxo-1,2,3,4-tetrahydro-pyrimido[4,5-b]quinolin-3-ium (4)**

2-Amino-1H-quinolin-4-one **1** (518,8 mg, 3.2 mmol) was dissolved in DMF (20 mL) followed by addition of paraformaldehyde (137.3 mg, 4.6 mmol) and dimethylamine (1 mL, 7.9 mmol, 40% aqueous solution). The mixture was stirred at 50°C for 12 hours. The solvent was partly evaporated in vacuum. The residual was diluted with water and the resulting suspension was filtered off. The filtrate was left to stand at room temperature overnight. The precipitated product **4** was isolated by filtration, washed with water and dried. Yield: 237 mg (18%).

^1^H NMR (400 MHz, DMSO-d6) δ ppm 14.72 (s, 1H), 11.53 (br s, 1H), 8.18 (dd, J = 8.0, 1.0 Hz, 1H), 8.01 (dd, J = 8.0, 1.2 Hz, 1H), 7.73 (br d, J = 8.0 Hz, 1H), 7.65 (ddd, J = 8.0, 7.0, 1.2 Hz, 1H), 7.60 (ddd, J = 8.2, 7.0, 1.0 Hz, 1H), 7.42 (br d, J = 8.2 Hz, 1H), 7.32–7.23 (m, 2H), 7.01 (br s, 2H), 5.02 (s, 2H), 4.80 (s, 2H), 4.36 (s, 2H), 3.22 (s, 6H).

^13^C NMR (101 MHz, DMSO-d6) δ ppm 175.0, 173.2, 154.0, 146.4, 138.7, 137.5, 131.8, 131.4, 125.0, 124.4, 122.8, 122.5, 122.3, 121.4, 117.7, 116.6, 97.7, 91.0, 74.3, 59.2, 48.3, 44.6.

HRMS (ESI): m/z calcd for [C_23_H_24_N_5_O_2_]+ 402.1925; found 402.1923.

#### General synthesis method of compounds 5a-c

2-Amino-1H-quinolin-4-one (250 mg, 1.6 mmol) was dissolved in ethanol (5 mL) and followed by addition of paraformaldehyde (93.7 mg, 3.2 mmol) and primary alkylamine (1.6 mmol). Reaction mixture was refluxed for 6 hours. The precipitate was filtered, washed with cold ethanol and dried.

**3-(5-Oxo-1,2-dihydropyrimido[4,5-b]quinolin-3(4H,5H,10H)-yl)propanoic acid (5a)**:

Yield: 418 mg (98%).

^1^H NMR (400 MHz, DMSO-d6) δ ppm 12.05 (br s, 1H), 10.82 (br s, 1H), 7.93 (dd, J = 8.1, 1.5 Hz, 1H), 7.44 (ddd, J = 8.4, 7.0, 1.5 Hz, 1H), 7.29 (br d, J = 8.4 Hz, 1H), 7.11 (ddd, J = 8.1, 7.0, 1.1 Hz, 1H), 6.52 (t, J = 3.2 Hz, 1H), 4.01 (d, J = 3.2 Hz, 2H), 3.58 (s, 2H), 2.70 (t, J = 7.1 Hz, 2H), 2.43 (t, J = 7.1 Hz, 2H).

^13^C NMR (101 MHz, DMSO-d6) δ ppm 173.4, 172.6, 148.5, 137.9, 130.0, 124.5, 122.8, 121.1, 116.2, 94.0, 61.5, 48.0, 47.4, 32.9.

HRMS (ESI): m/z calcd for [C_14_H_15_N_3_O_3_ + H]+ 274.1186; found 274.1187.

**3-(1-Phenylethyl)-1,2,3,4-tetrahydropyrimido[4,5-b]quinolin-5(10H)-one (5b)**:

Yield: 419 mg (88%).

^1^H NMR (300 MHz, DMSO-d6) δ ppm 10.89 (s, 1H), 7.92 (dd, J = 8.0, 1.2 Hz, 1H), 7.44 (ddd, J = 8.4, 7.0, 1.2 Hz, 1H), 7.38–7.19 (m, 6H), 7.11 (ddd, J = 8.0, 7.0, 1.0 Hz, 1H), 6.55 (br dd, J = 3.0, 2.6 Hz, 1H), 4.08 (dd, J = 11.2, 3.0 Hz, 1H), 3.96 (dd, J = 11.2, 2.6 Hz, 1H), 3.66–3.54 (m, 3H), 1.32 (d, J = 6.6 Hz, 3H).

^13^C NMR (75 MHz, DMSO-d6) δ ppm 172.4, 148.9, 144.9, 137.9, 130.0, 128.3, 127.1, 126.9, 124.5, 122.9, 121.1, 116.2, 94.3, 59.5, 58.8, 45.5, 21.4.

HRMS (ESI): m/z calcd for [C_19_H_19_N_3_O + H]^+^ 306.1601; found 306.1599.

**3-Propyl-1,2,3,4-tetrahydropyrimido[4,5-b]quinolin-5(10H)-one (5c)**:

Yield: 323 mg (85%)

^1^H NMR (300 MHz, DMSO-d6) δ ppm 10.87 (br s, 1H), 7.94 (dd, J = 8.0, 1.3 Hz, 1H), 7.43 (ddd, J = 8.2, 7.1, 1.3 Hz, 1H), 7.28 (br d, J = 8.0 Hz, 1H), 7.11 (ddd, J = 8.0, 7.1, 1.0 Hz, 1H), 6.56 (br t, J = 2.1 Hz, 1H), 3.98 (d, J = 2.1 Hz, 2H), 3.55 (s, 2H), 2.39 (t, J = 7.3 Hz, 2H), 1.48 (m, 2H), 0.86 (t, J = 7.3 Hz, 3H).

^13^C NMR (75 MHz, DMSO-d6) δ ppm 172.6, 148.7, 137.9, 130.0, 124.5, 122.9, 121.2, 116.2, 94.3, 61.8, 54.2, 47.4, 20.5, 11.8.

HRMS (ESI): m/z calcd for [C_14_H_17_N_3_O + H]^+^ 244.1444; found 244.1446.

**Preparation of 2-amino-3-(((3-((2-amino-4-oxo-1**,**4-dihydroquinolin-3-yl)methyl)-4-oxo-1,4-dihydroquinolin-2-yl)amino)methyl)quinolin-4(1H)-one (6)**

Paraformaldehyde (800 mg, 26.6 mmol) was added to the solution of 2-amino-1H-quinolin-4-one **1** (1 g, 6.24 mmol) in DMF (40 mL) and the reaction mixture was stirred at 90°C for 3.5 hours. After cooling to room temperature EtOAc (25 mL)was added. The resulting suspension was filtered and washed with EtOAc. The filtrate was diluted with water to obtain a precipitate and two-phase system. The precipitate was filtered and washed thoroughly with water. The dry residual material was dissolved in MeCN and purified by semipreparative HPLC. Yield: 242 mg (23%).

NMR measurement at +25°C:

^1^H NMR (600.2 MHz, DMF-d7) δ ppm 12.98 (s, 1H), 10.81 (t, J = 6.2 Hz, 1H), 8.21 (dd, J = 8.0, 1.2 Hz, 1H), 8.18 (dd, J = 8.1, 1.2 Hz, 1H), 8.11 (dd, J = 8.1, 1.2 Hz, 1H), 7.62 (d, J = 8.3 Hz, 1H), 7.50 (ddd, J = 8.2, 7.8, 1.2 Hz, 1H), 7.49 (ddd, J = 8.3, 7.8, 1.2 Hz, 1H), 7.48 (ddd, J = 8.3, 7.8, 1.2 Hz, 1H), 7.36 (d, J = 8.2 Hz, 1H), 7.31 (d, J = 8.3 Hz, 1H), 7.23 (dd, J = 8.1, 7.8 Hz, 1H), 7.21 (dd, J = 8.0, 7.8 Hz, 1H), 7.15 (dd, J = 8.1, 7.8 Hz, 1H), 6.95 (s, 2H), 4.81 (br, 1H), 4.29 (br, 1H), 3.98 (br d, J = 14.5 Hz, 1H), 3.80 (br d, J = 14.5 Hz, 1H).

^13^C NMR (150.9 MHz, DMF-d7) δ ppm 175.50, 174.27, 172.96, 155.68, 154.85, 153.16, 138.51, 138.34, 137.87, 131.18, 130.31, 129.72, 125.14, 125.10, 124.96, 123.36, 122.54, 122.52, 122.42, 122.18, 121.61, 116.92, 116.87, 116.39, 103.15, 101.40, 99.85, 35.30, 18.73.

NMR measurement at -40°C:

^1^H NMR (600.2 MHz, DMF-d7) δ ppm 13.14 (br s, 1H), 12.35 (br s, 1H), 12.31 (br s, 1H), 10.95 (s, 1H), 9.70 (br s, 1H), 8.19 (br d, J = 7.8 Hz, 1H), 8.18 (br d, J = 7.8 Hz, 1H), 8.10 (br d, J = 7.8 Hz, 1H), 7.64 (d, J = 7.8 Hz, 1H), 7.55 (dd, J = 7.8, 7.8 Hz, 1H), 7.55 (dd, J = 8.3, 7.8 Hz, 1H), 7.48 (br dd, J = 7.8, 7.8 Hz, 1H), 7.39 (d, J = 8.3 Hz, 1H), 7.34 (br d, J = 7.8 Hz, 1H), 7.252 (dd, J = 7.8, 7.8 Hz, 1H), 7.246 (dd, J = 7.8, 7.8 Hz, 1H),7.23 (br s, 1H), 7.22 (br, 2H), 7.20 (dd, J = 7.8, 7.8 Hz, 1H), 4.82 (br, 1H), 4.28 (br, 1H), 3.97 (d, J = 14.7 Hz, 1H), 3.79 (d, J = 14.7 Hz, 1H).

^13^C NMR (150.9 MHz, DMF-d7) δ ppm 175.43, 174.11, 172.82, 155.79, 154.96, 153.21, 138.54, 138.42, 138.01, 131.58, 130.70, 130.17, 125.25, 123.31, 122.76, 122.52, 122.45, 122.42, 122.03, 117.19, 117.09, 116.61, 103.21, 101.49, 99.93, 35.62, 18.86.

HRMS (ESI): m/z calcd for [C_29_H_24_N_6_O_3_ + H]^+^ 505.1983; found 505.1982.

**Preparation of 3-((1H-indol-3-yl)methyl)-2-aminoquinolin-4(1H)**-**one (7)**

The compound **2a** (50 mg, 0.2 mmol) and indole (234 mg, 2.0 mmol) were dissolved in DMF (5 mL) and the reaction mixture was stirred and heated at 90°C for 6 hours. The reaction mixture was cooled to room temperature and then diluted with water. The resulting suspension was extracted with EtOAc (2x 10 mL). The combined organic layers were washed with water, dried Na2SO4 and evaporated under low pressure. The dry residual material was dissolved in MeCN and purified by semipreparative HPLC. Yield: 23 mg (35%).

^1^H NMR (400 MHz, DMSO-d6) δ ppm 10.78 (br s, 1H), 10.61 (s, 1H), 8.04 (dd, J = 8.0, 1.3 Hz, 1H), 7.71 (br d, J = 8.0 Hz, 1H), 7.42 (ddd, J = 8.3, 7.0, 1.3 Hz, 1H), 7.26 (br d, J = 8.1 Hz, 1H), 7.23 (br d, J = 8.3 Hz, 1H), 7.16–7.09 (m, 2H), 6.98 (ddd, J = 8.1, 7.1, 0.9 Hz, 1H), 6.85 (ddd, J = 8.0, 7.1, 0.8 Hz, 1H), 5.88 (s, 2H), 3.90 (s, 2H).

^13^C NMR (101 MHz, DMSO-d6) δ ppm 173.5, 151.5, 137.5, 136.4, 129.8, 127.4, 125.2, 122.7, 122.6, 121.1, 120.6, 119.7, 117.8, 116.0, 113.9, 111.0, 100.1, 18.4.

HRMS (ESI): m/z calcd for [C_18_H_15_N_3_O + H]^+^ 290.1288; found 290.1288.

**Preparation of 2-amino-3-((phenylthio)methyl)quinolin-4(1H)**-**one (8)**

The compound 2a (50 mg, 0.2 mmol) and thiophenol (204 μL, 2.0 mmol) were dissolved in DMF (5 mL) and the reaction mixture was stirred and heated at 90°C for 3 hours. The reaction mixture was cooled to room temperature and then diluted with water. The resulting precipitate was filtered and washed with water. Yield: 51 mg (78%).

^1^H NMR (400 MHz, DMSO-d6) δ ppm 10.70 (s, 1H), 7.96 (dd, J = 8.0, 1.5 Hz, 1H), 7.46 (ddd, J = 8.4, 7.1, 1.5 Hz, 1H), 7.41–7.36 (m, 2H), 7.31–7.24 (m, 3H), 7.17–7.08 (m, 2H), 6.25 (s, 2H), 4.25 (s, 2H).

^13^C NMR (101 MHz, DMSO-d6) δ ppm 173.4, 152.0, 138.7, 137.5, 130.4, 128.7, 127.4, 125.0, 124.7, 122.2, 121.5, 116.1, 94.7, 26.6.

HRMS (ESI): m/z calcd for [C_16_H_14_N_2_OS + H]^+^ 283.0900; found 283.0898.

## Supporting information

S1 Fig ^1^H NMR spectrum of compound 2a in DMSO-*d*_*6*_ (400 MHz). (TIF)

S2 Fig ^13^C NMR spectrum of compound 2a in DMSO-*d*_*6*_ (101 MHz). (TIF)

S3 Fig ^1^H NMR spectrum of compound 2b in DMSO-*d*_*6*_ (400 MHz). (TIF)

S4 Fig ^13^C NMR spectrum of compound 2b in DMSO-*d*_*6*_ (101MHz). (TIF)

S5 Fig ^1^H NMR spectrum of compound 2c in DMSO-*d*_*6*_ (300 MHz). (TIF)

S6 Fig ^13^C NMR spectrum of compound 2c in DMSO-*d*_*6*_ (75 MHz). (TIF)

S7 Fig ^1^H NMR spectrum of compound 3 in DMSO-*d*_*6*_ (400 MHz). (TIF)

S8 Fig ^13^C NMR spectrum of compound 3 in DMSO-*d*_*6*_ (101 MHz). (TIF)

S9 Fig ^1^H NMR spectrum of compound 4 in DMSO-*d*_*6*_ (400 MHz). (TIF)

S10 Fig ^13^C NMR spectrum of compound 4 in DMSO-*d*_*6*_ (101 MHz). (TIF)

S11 Fig ^1^H-^1^H COSY spectrum of compound 4 in DMSO-*d*_*6*_ (400 MHz). (TIF)

S12 Fig ^1^H-^13^C HMBC spectrum of compound 4 in DMSO-*d*_*6*_ (400/101 MHz). (TIF)

S13 Fig ^1^H-^13^C HMQC spectrum of compound 4 in DMSO-*d*_*6*_ (HMQC, 400/101 MHz). (TIF)

S14 Fig ^1^H NMR spectrum of compound 5a in DMSO-*d*_*6*_ (400 MHz). (TIF)

S15 Fig ^13^C NMR spectrum of compound 5a in DMSO-*d*_*6*_ (101 MHz). (TIF)

S16 Fig ^1^H NMR spectrum of compound 5b in DMSO-*d*_*6*_ (300 MHz). (TIF)

S17 Fig ^13^C NMR spectrum of compound 5b in DMSO-*d*_*6*_ (75 MHz). (TIF)

S18 Fig ^1^H NMR spectrum of compound 5c in DMSO-*d*_*6*_ (300 MHz). (TIF)

S19 Fig ^13^C NMR spectrum of compound 5c in DMSO-*d*_*6*_ (75 MHz). (TIF)

S20 Fig ^1^H NMR spectrum of compound 6 in DMF-*d*_*7*_ (600 MHz). (TIF)

S21 Fig ^13^C NMR spectrum of compound 6 in DMF-*d*_*7*_ (151 MHz). (TIF)

S22 Fig ^1^H NMR spectrum of compound 7 in DMSO-*d*_*6*_ (400 MHz). (TIF)

S23 Fig ^13^C NMR spectrum of compound 7 in DMSO-*d*_*6*_ (101 MHz). (TIF)

S24 Fig ^1^H NMR spectrum of compound 8 in DMSO-*d*_*6*_ (400 MHz). (TIF)

S25 Fig ^13^C NMR spectrum of compound 8 in DMSO-*d*_*6*_ (101 MHz). (TIF)

S26 Fig HRMS spectrum of compound 2a. (TIF)

S27 Fig HRMS spectrum of compound 2b. (TIF)

S28 Fig HRMS spectrum of compound 2c. (TIF)

S29 Fig HRMS spectrum of compound 3. (TIF)

S30 Fig HRMS spectrum of compound 4. (TIF)

S31 Fig HRMS spectrum of compound 5a. (TIF)

S32 Fig HRMS spectrum of compound 5b. (TIF)

S33 Fig HRMS spectrum of compound 5c. (TIF)

S34 Fig HRMS spectrum of compound 6. (TIF)

S35 Fig HRMS spectrum of compound 7. (TIF)

S36 Fig HRMS spectrum of compound 8. (TIF)

S37 Fig Proposed molecular structure of compound 6 and atom numbering used in the NMR structural analysis. (TIF)

S38 Fig Key long-range ^1^H-^13^C correlations observed at 25°C. All correlations of protons H-7, H-8, H-7’, H-8’, H-7” and H-8” are omitted for simplicity. Correlations of protons H-6, H-9, H-6’, H-9’, H-6” and H-9” to protonated-carbons of the same ring are also omitted for clarity.
(TIF)

S39 Fig Key ^1^H-^1^H NOE interactions observed in 6 at 25°C. (TIF)

S40 Fig Direct and long-range ^1^H-^15^N correlations observed in 6 at 25°C. (TIF)

S41 Fig Key long-range ^1^H-^13^C correlations observed at -40°C. All correlations of protons H-7, H-8, H-7’, H-8’, H-7” and H-8” are omitted for simplicity. Correlations of protons H-6, H-9, H-6’, H-9’, H-6” and H-9” to protonated-carbons of the same ring are also omitted for clarity.
(TIF)

S42 Fig Key ^1^H-^1^H NOE interactions observed in 6 at -40°C. (TIF)

S43 Fig Direct ^1^H-^15^N correlations observed in 6 at -50°C. (TIF)

S1 File NMR structure elucidation of 6. (DOCX)
