# Supplementary figures and images for: Study of 2-aminoquinolin-4(1H)-one under Mannich and retro-Mannich reaction
Source: PLoS One. 2017 May 30;12(5):e0175364. doi: 10.1371/journal.pone.0175364 (PMC5448738; doi:10.1371/journal.pone.0175364)

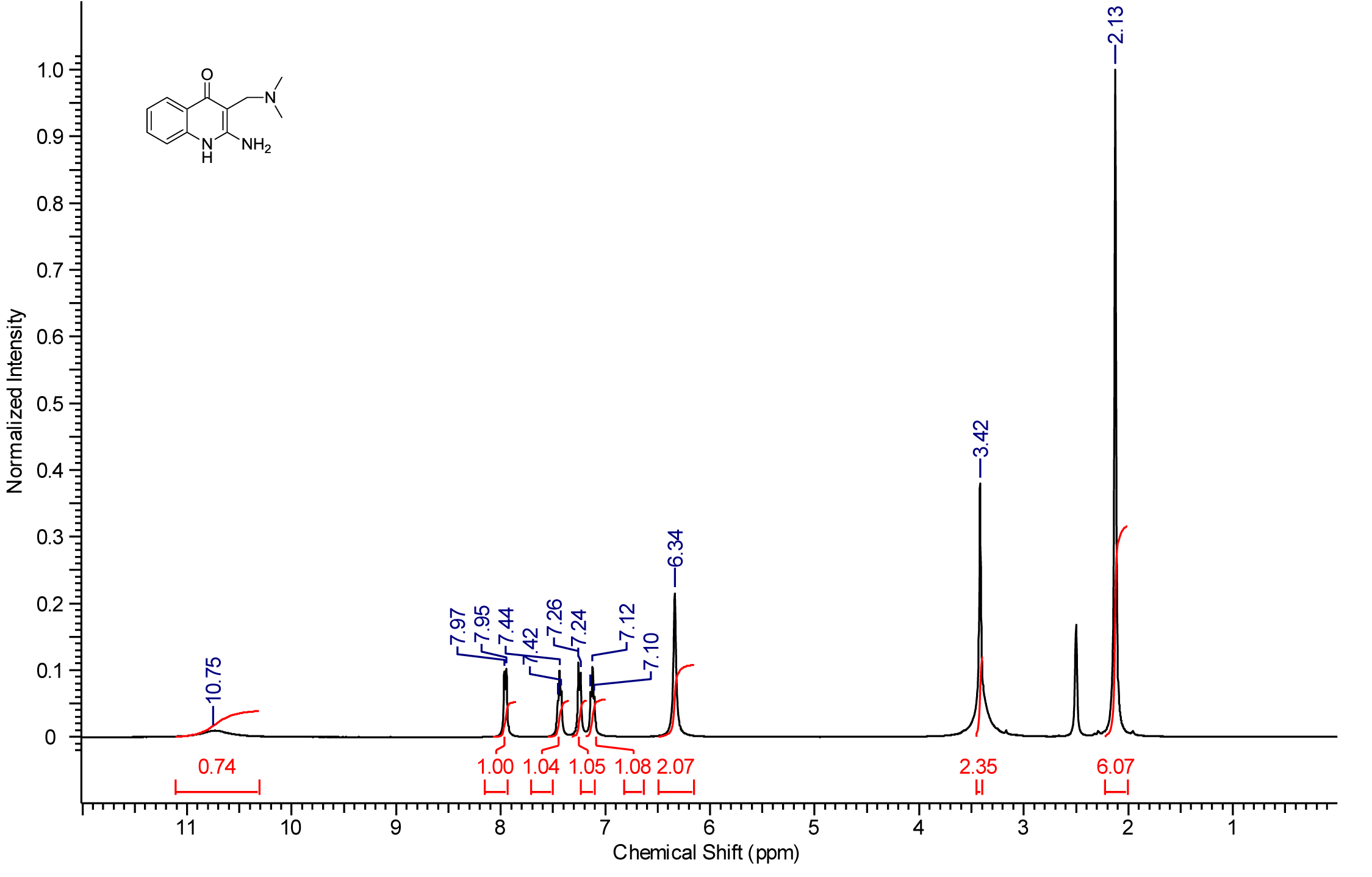

Supplement: S1 Fig — (TIF) [file pone.0175364.s001.tif]

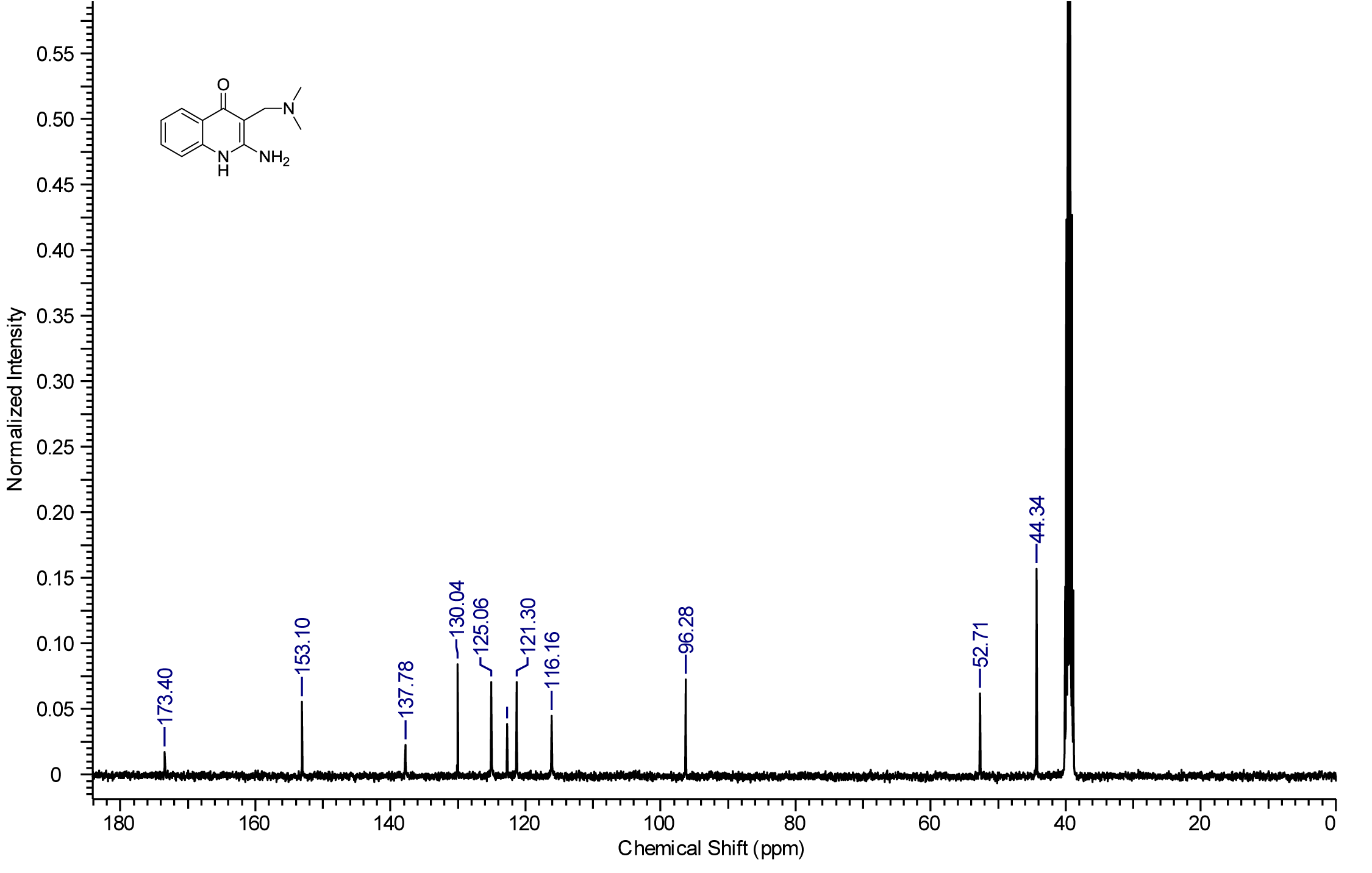

Supplement: S2 Fig — (TIF) [file pone.0175364.s002.tif]

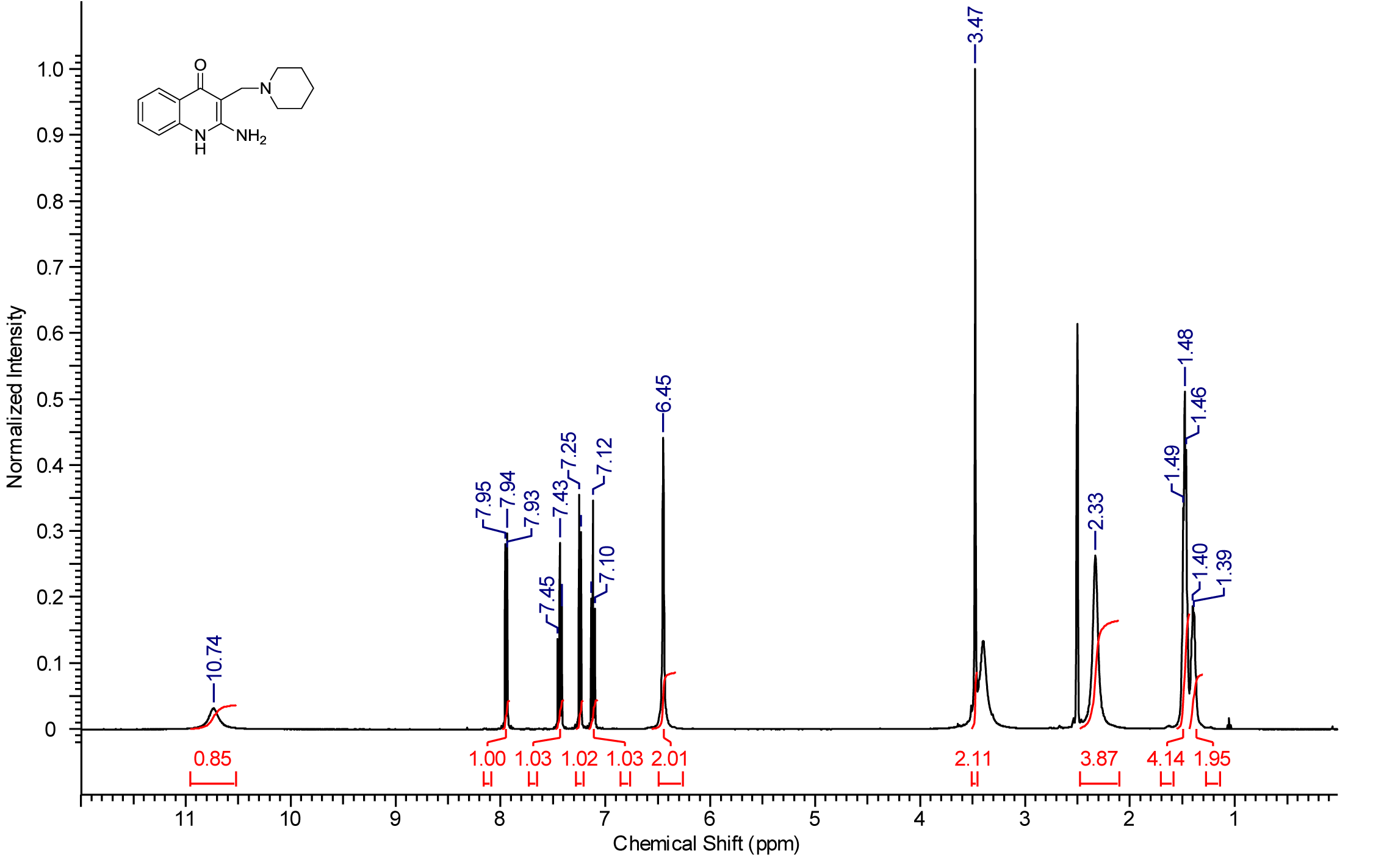

Supplement: S3 Fig — (TIF) [file pone.0175364.s003.tif]

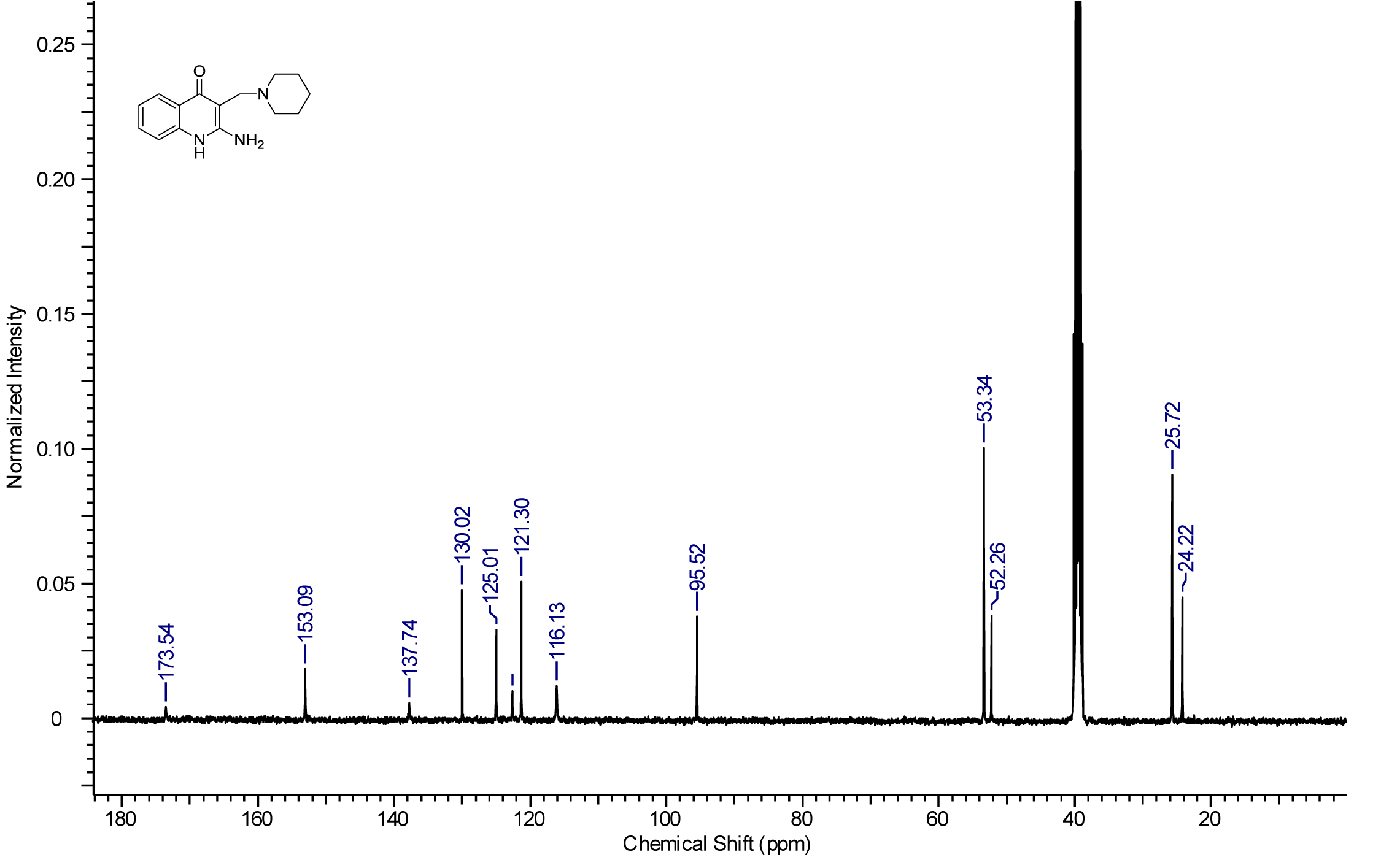

Supplement: S4 Fig — (TIF) [file pone.0175364.s004.tif]

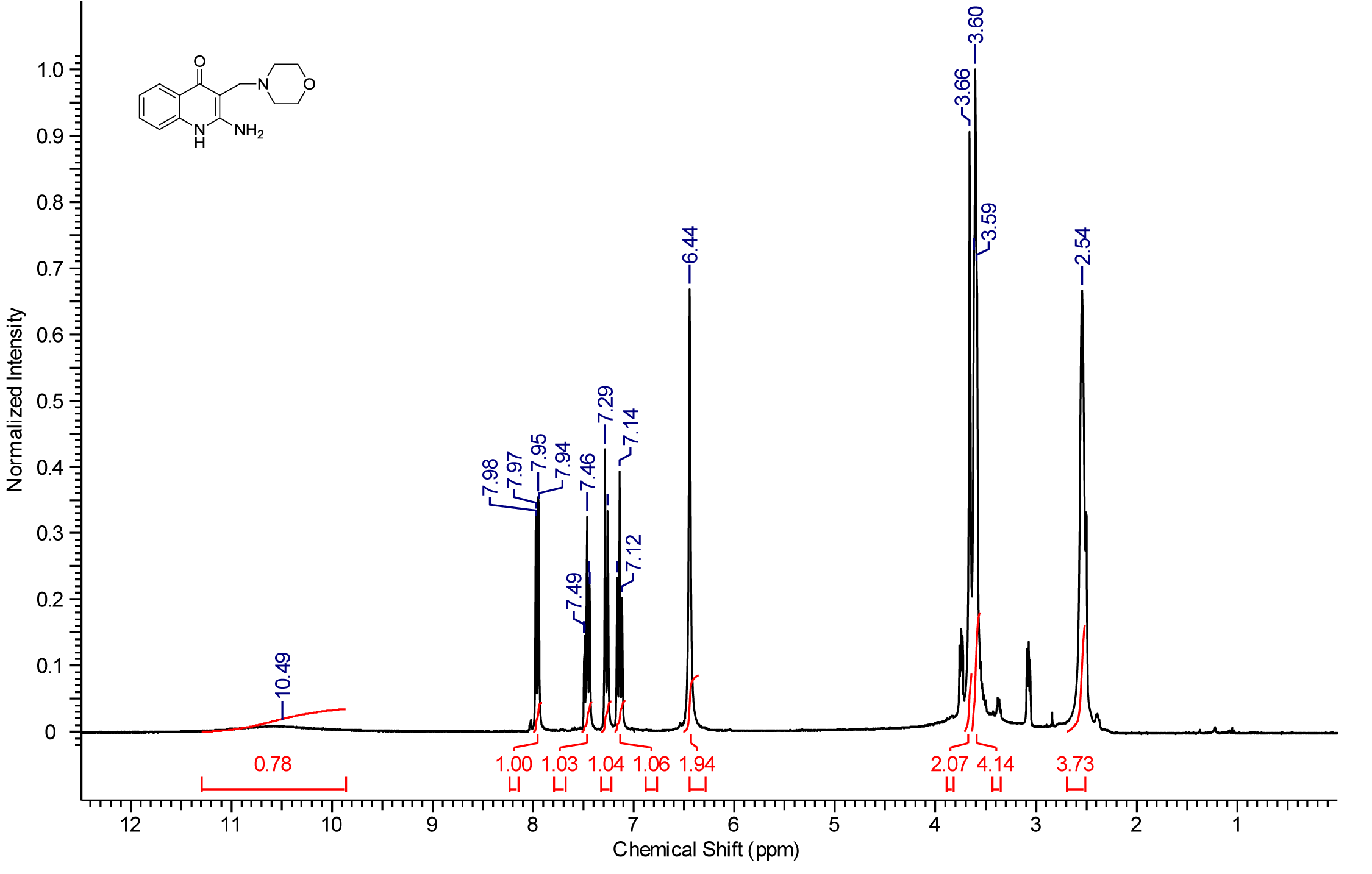

Supplement: S5 Fig — (TIF) [file pone.0175364.s005.tif]

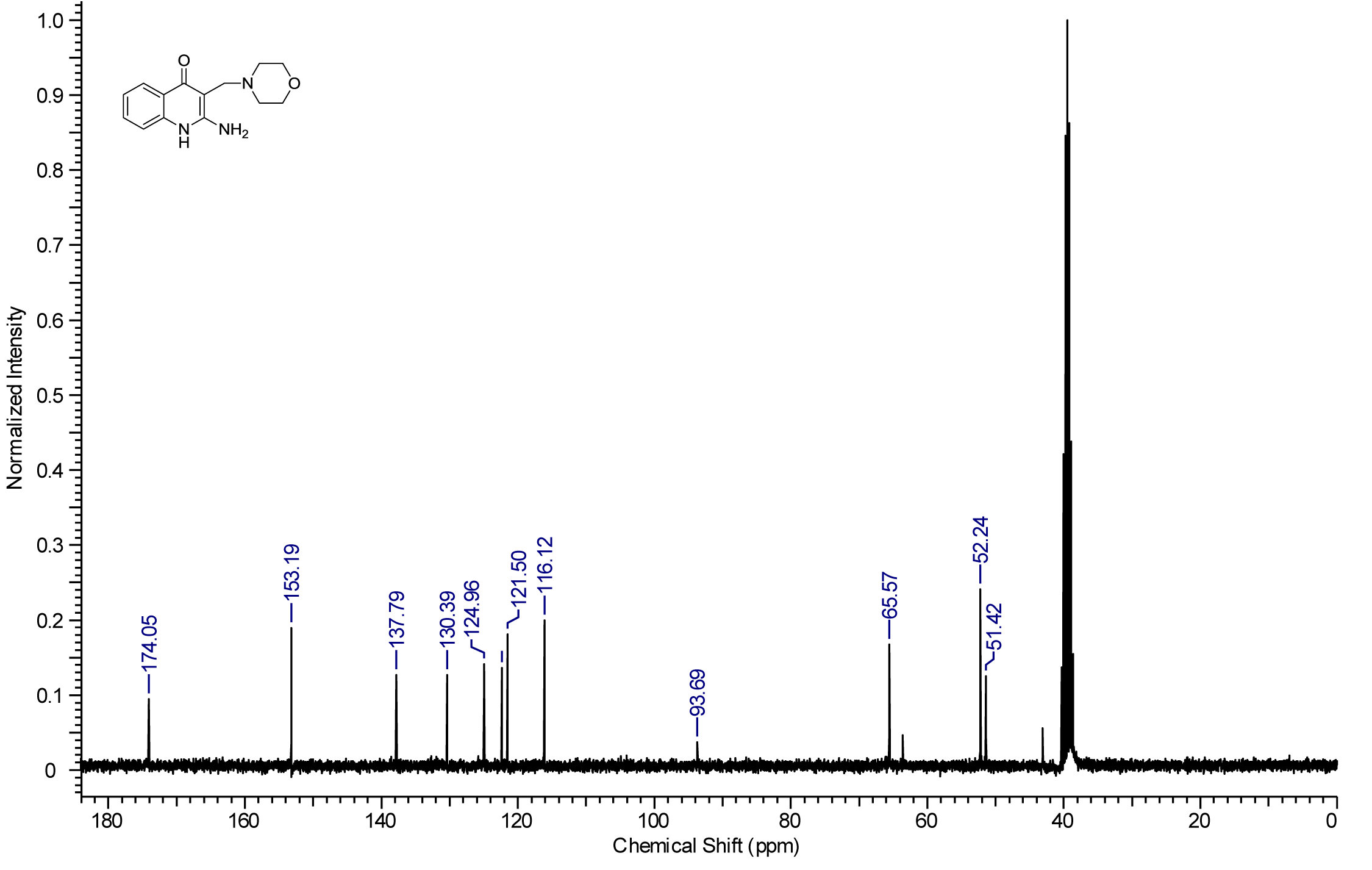

Supplement: S6 Fig — (TIF) [file pone.0175364.s006.tif]

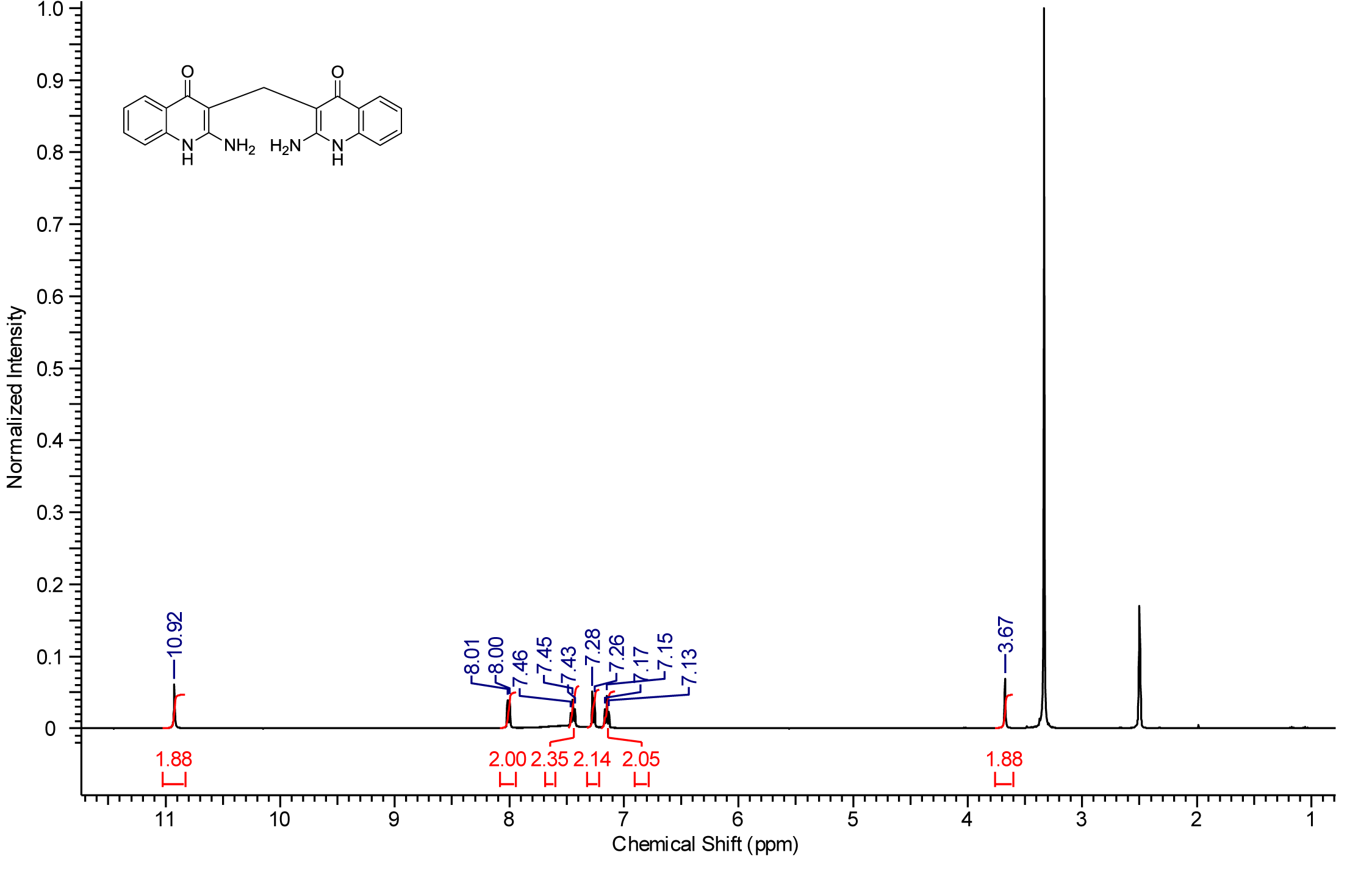

Supplement: S7 Fig — (TIF) [file pone.0175364.s007.tif]

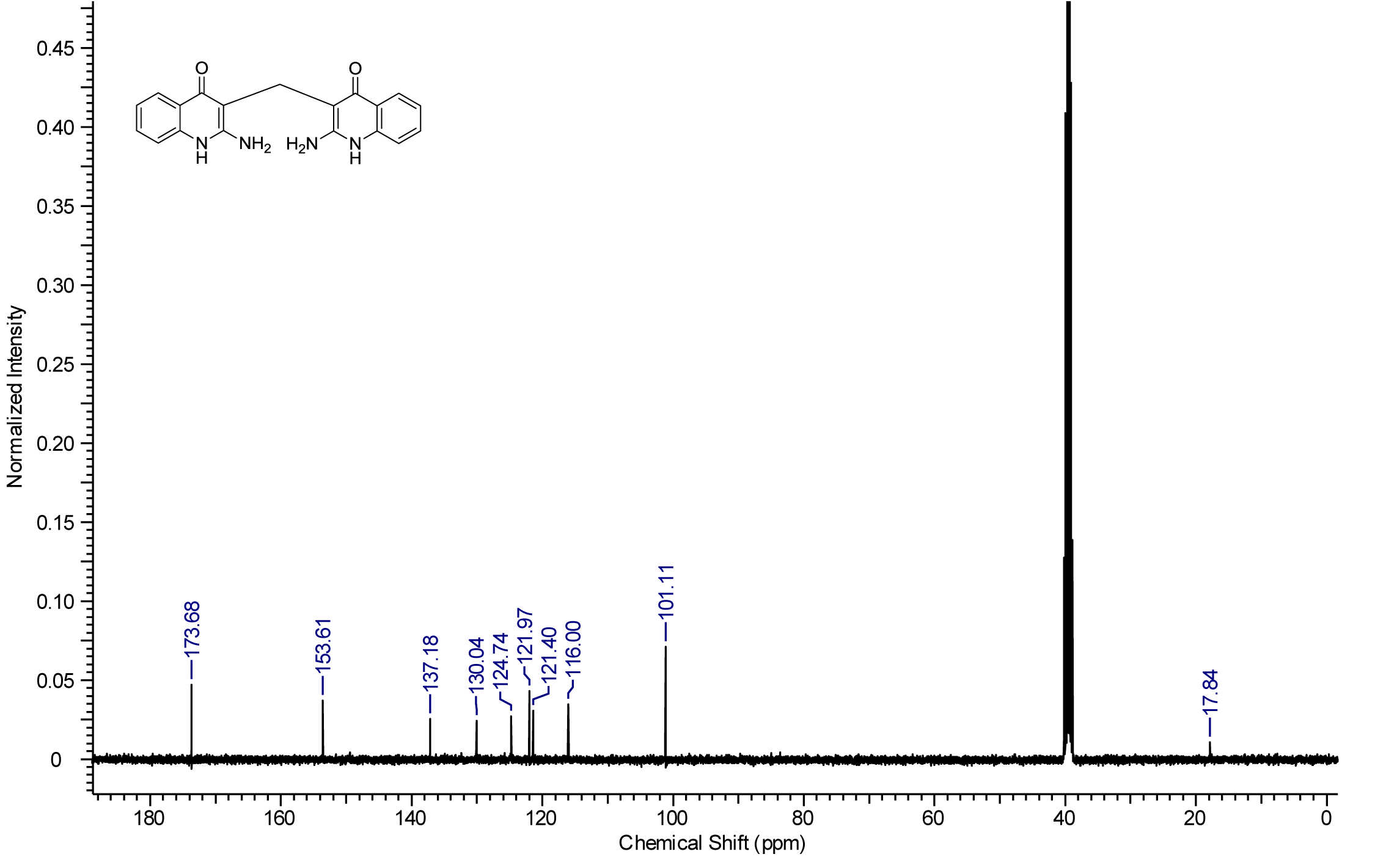

Supplement: S8 Fig — (TIF) [file pone.0175364.s008.tif]

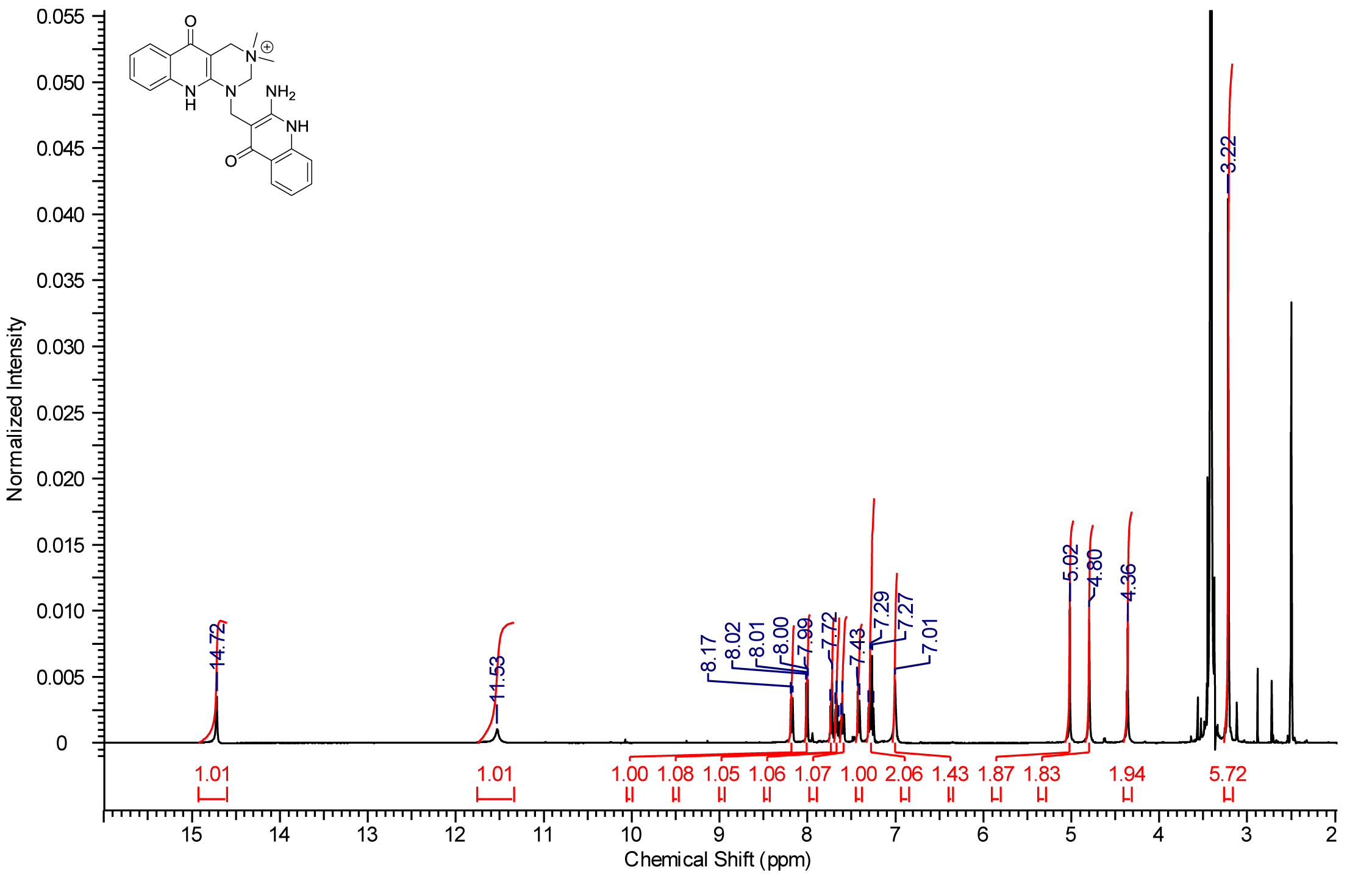

Supplement: S9 Fig — (TIF) [file pone.0175364.s009.tif]

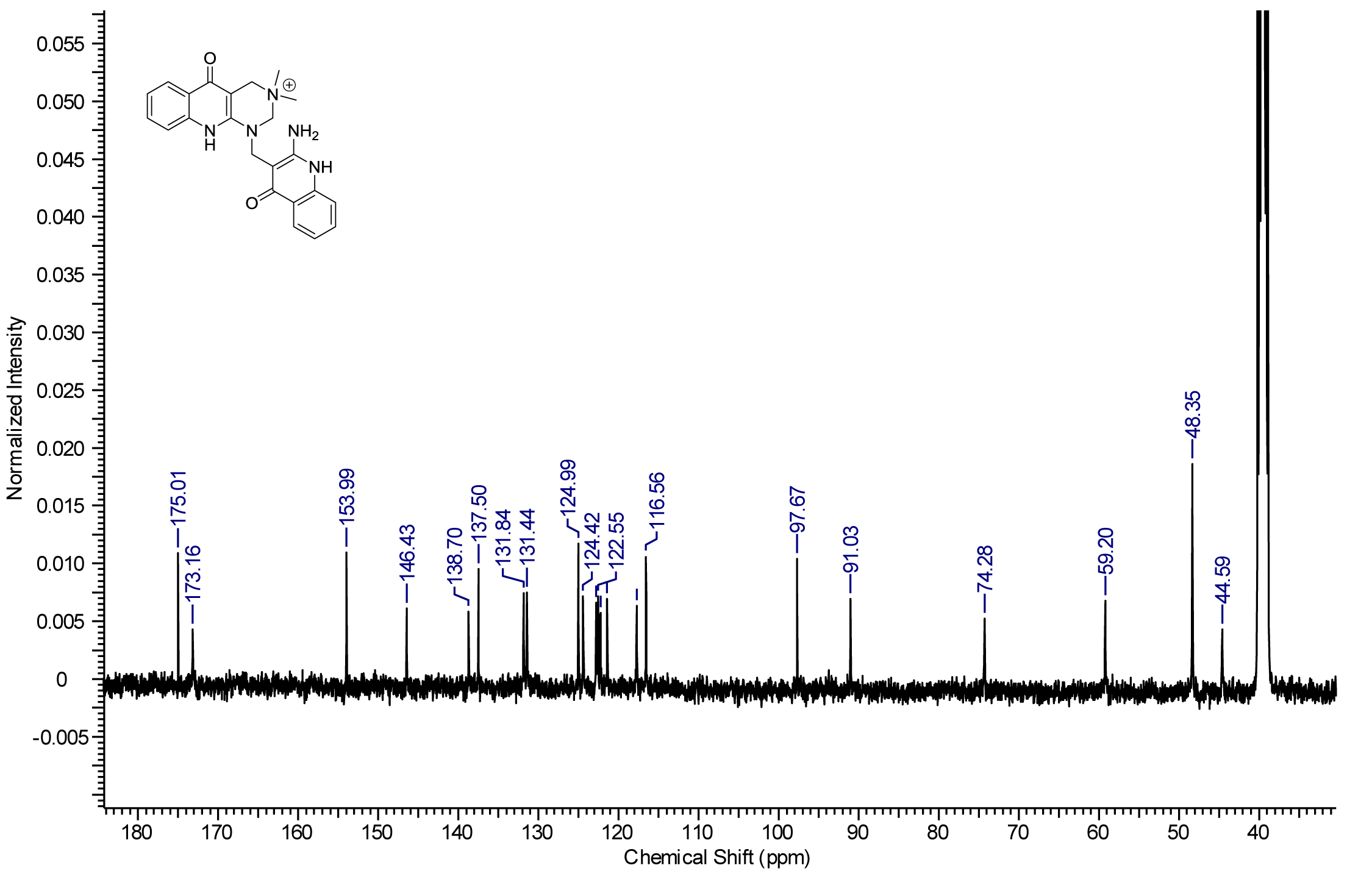

Supplement: S10 Fig — (TIF) [file pone.0175364.s010.tif]

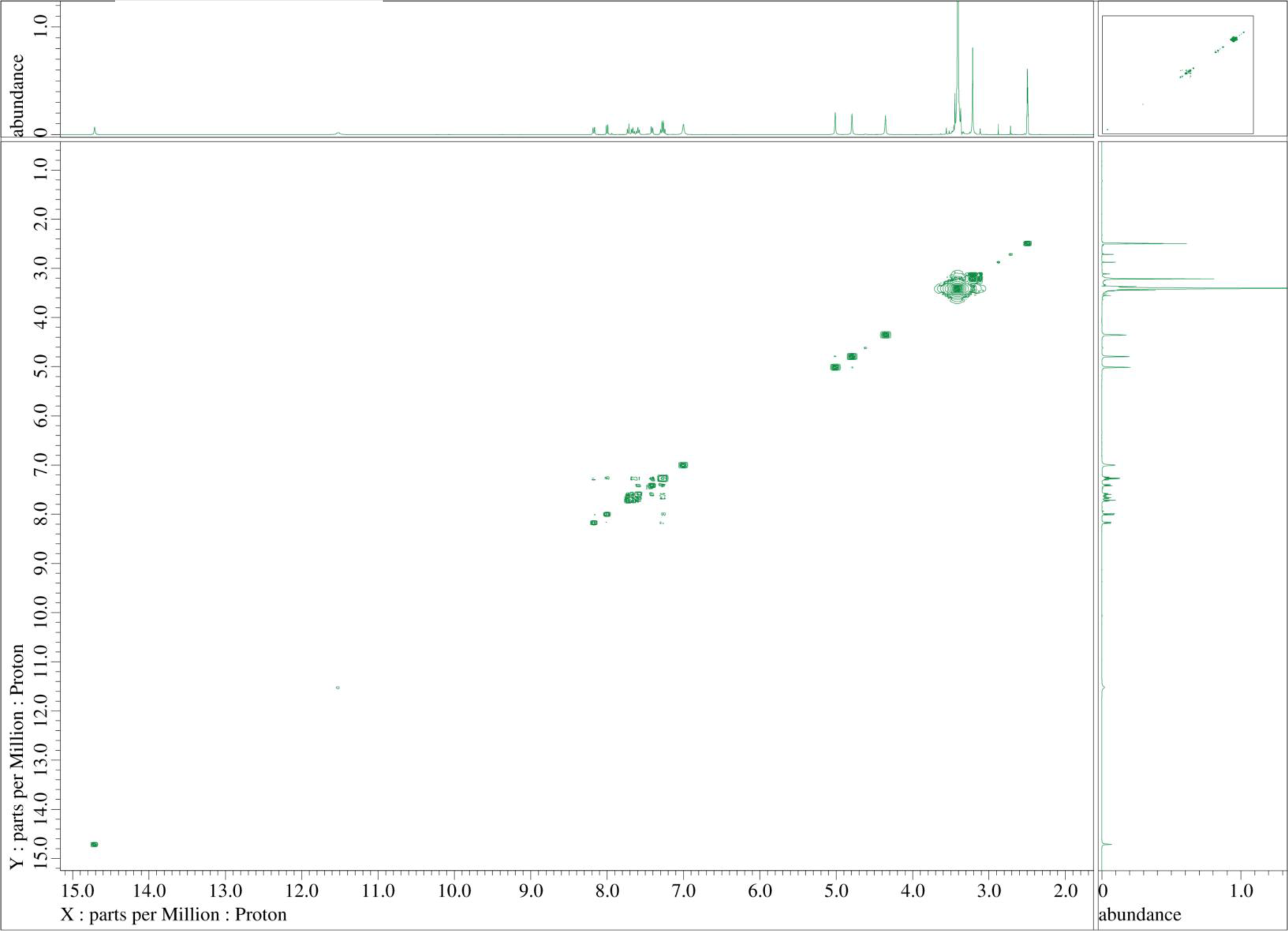

Supplement: S11 Fig — (TIF) [file pone.0175364.s011.tif]

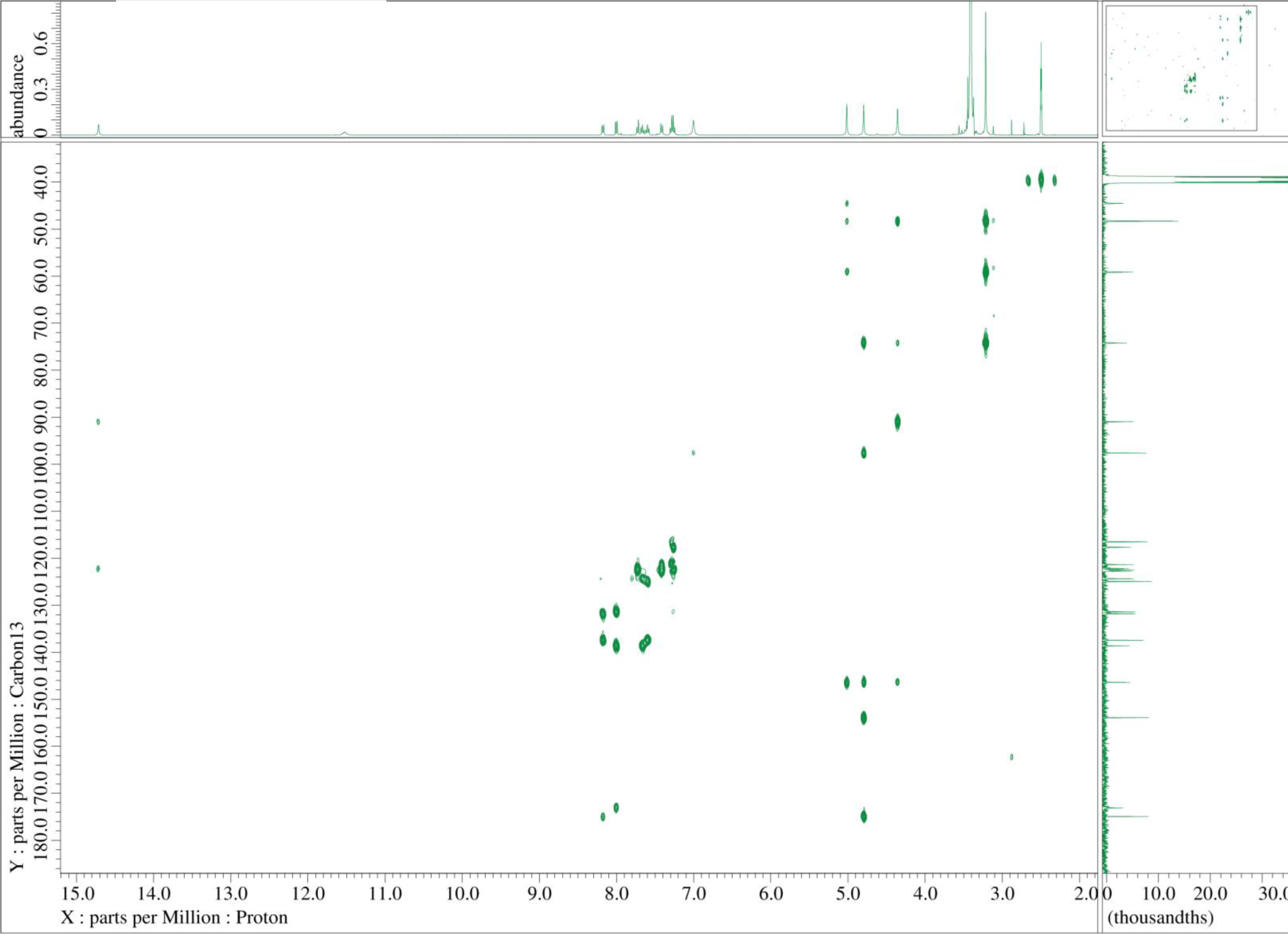

Supplement: S12 Fig — (TIF) [file pone.0175364.s012.tif]

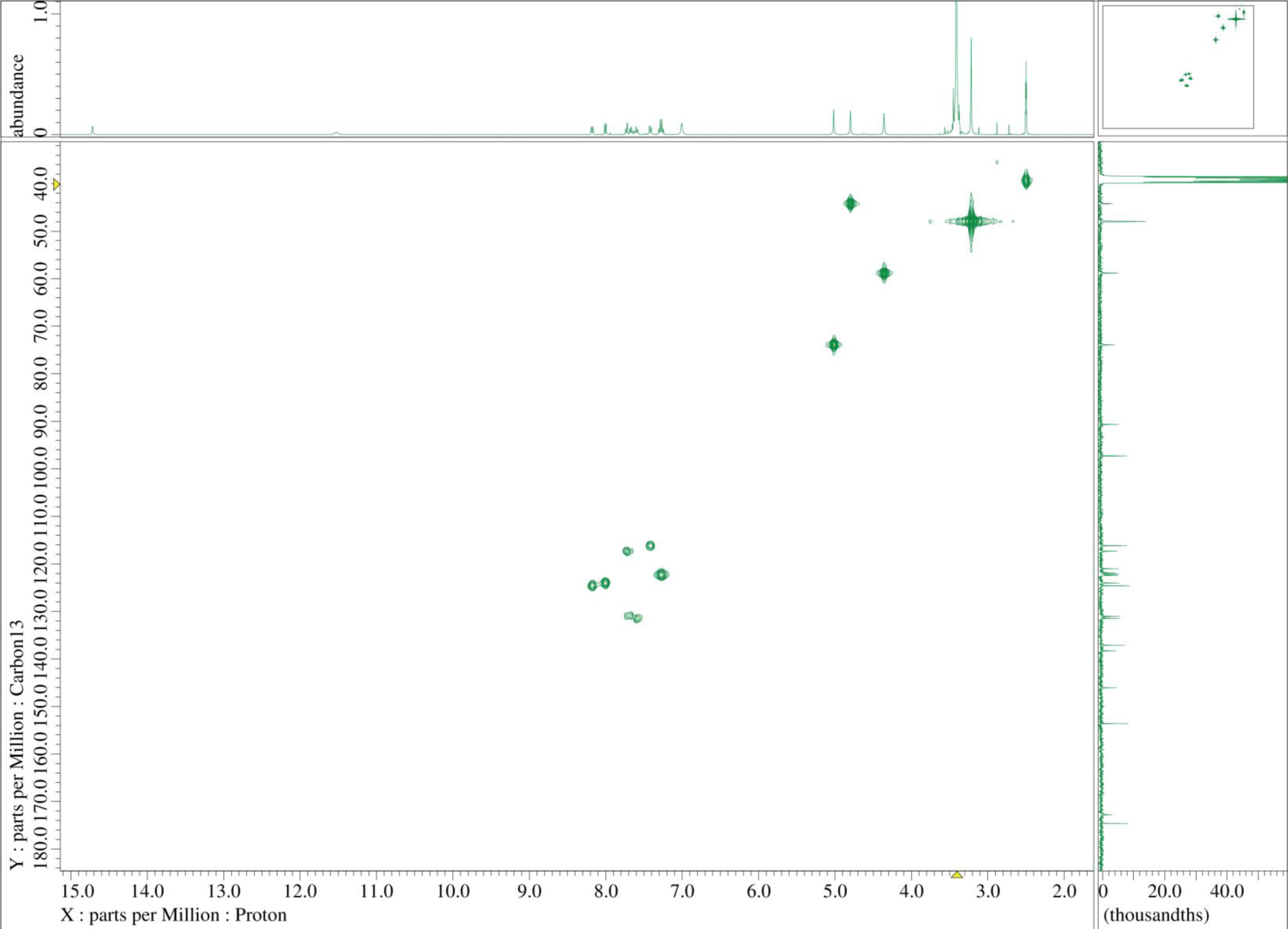

Supplement: S13 Fig — (TIF) [file pone.0175364.s013.tif]

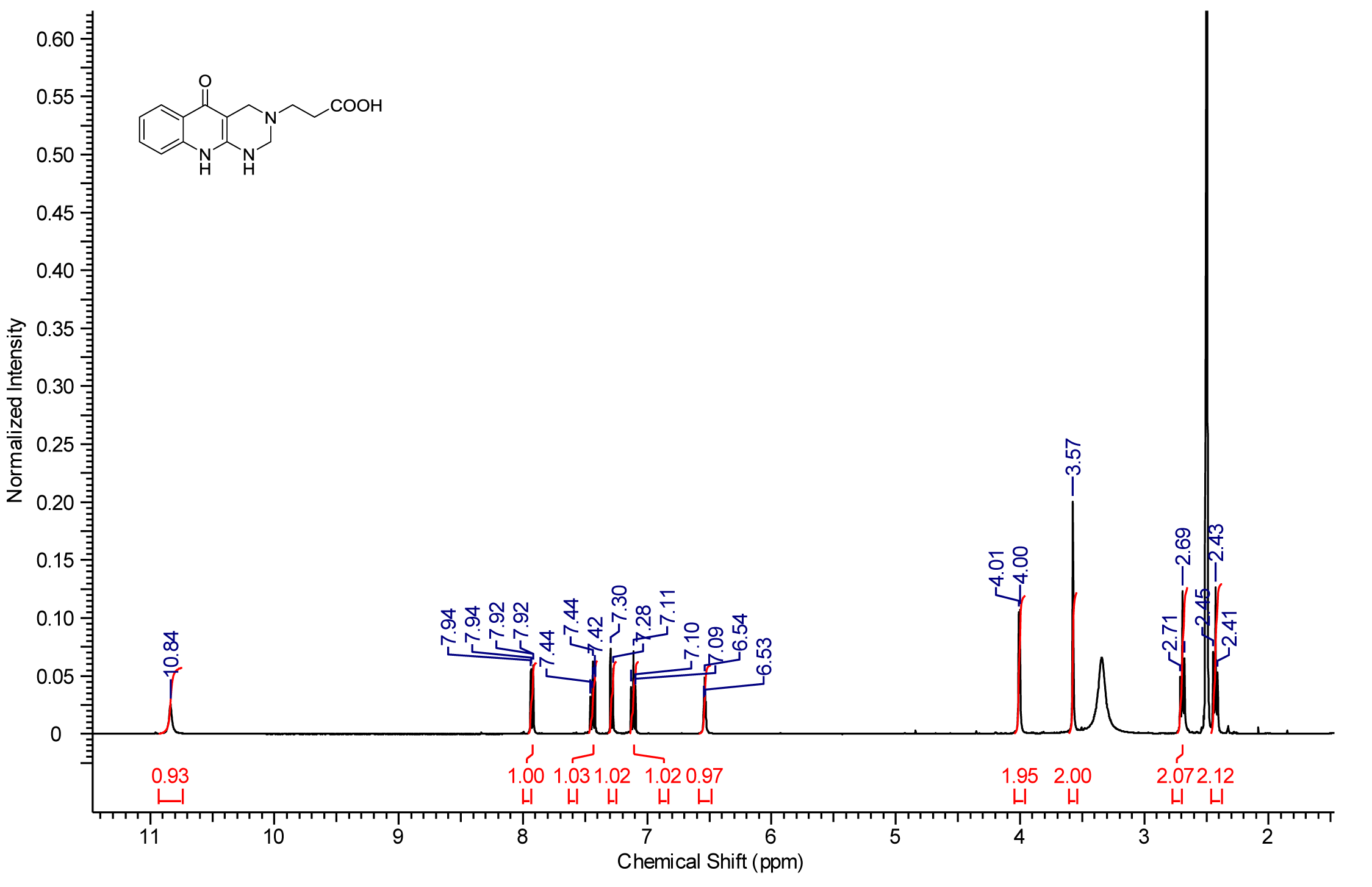

Supplement: S14 Fig — (TIF) [file pone.0175364.s014.tif]

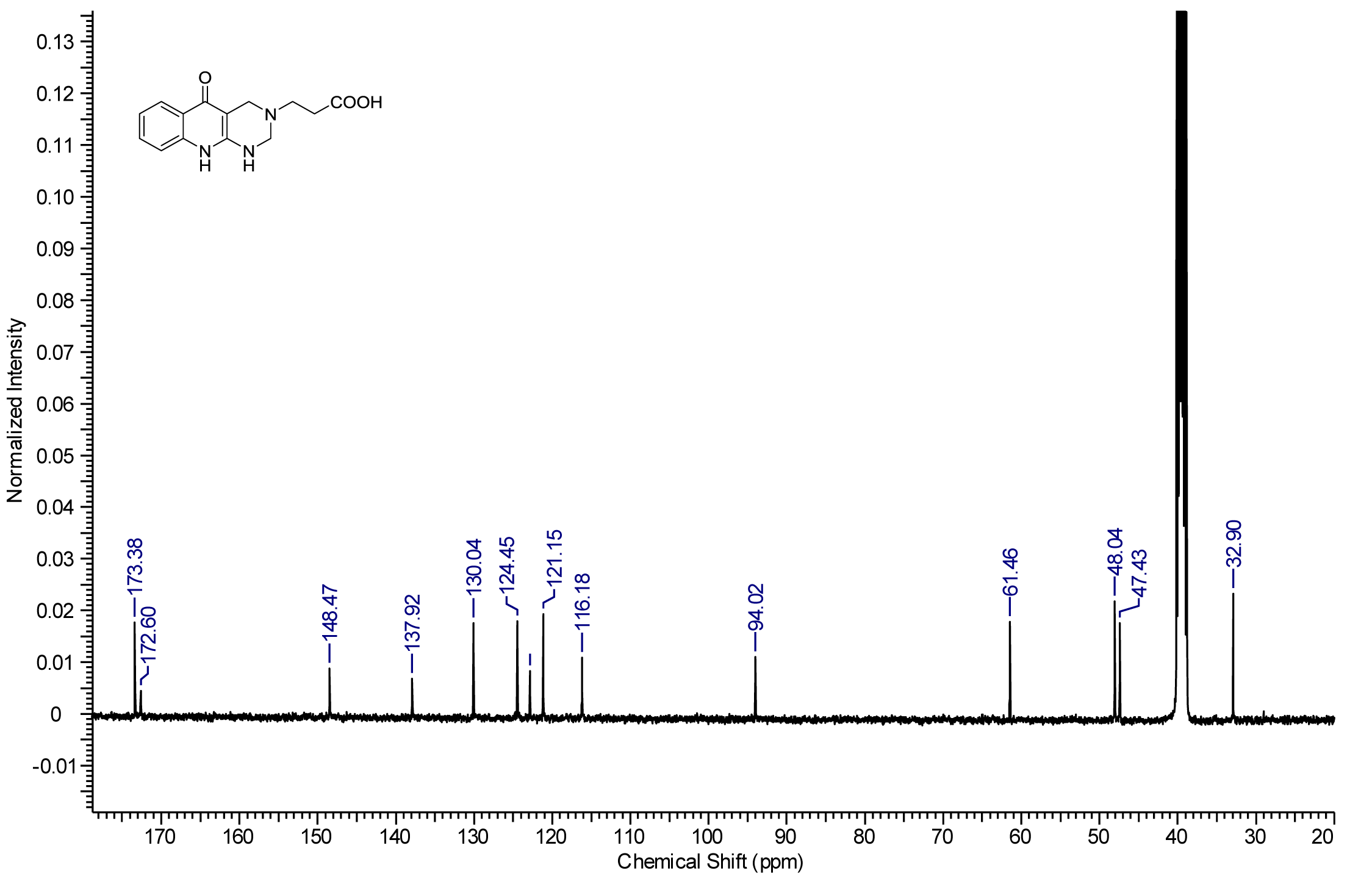

Supplement: S15 Fig — (TIF) [file pone.0175364.s015.tif]

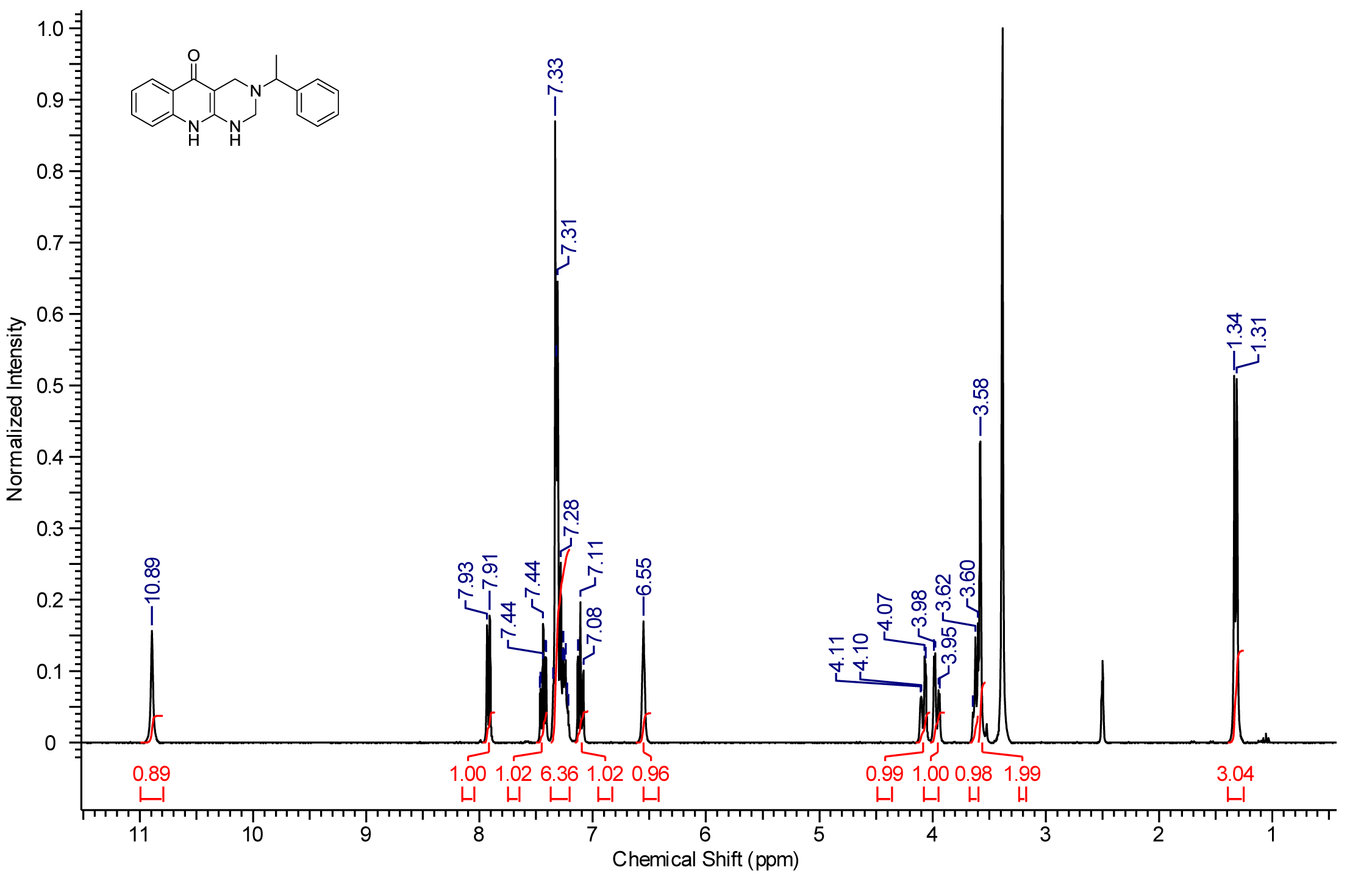

Supplement: S16 Fig — (TIF) [file pone.0175364.s016.tif]

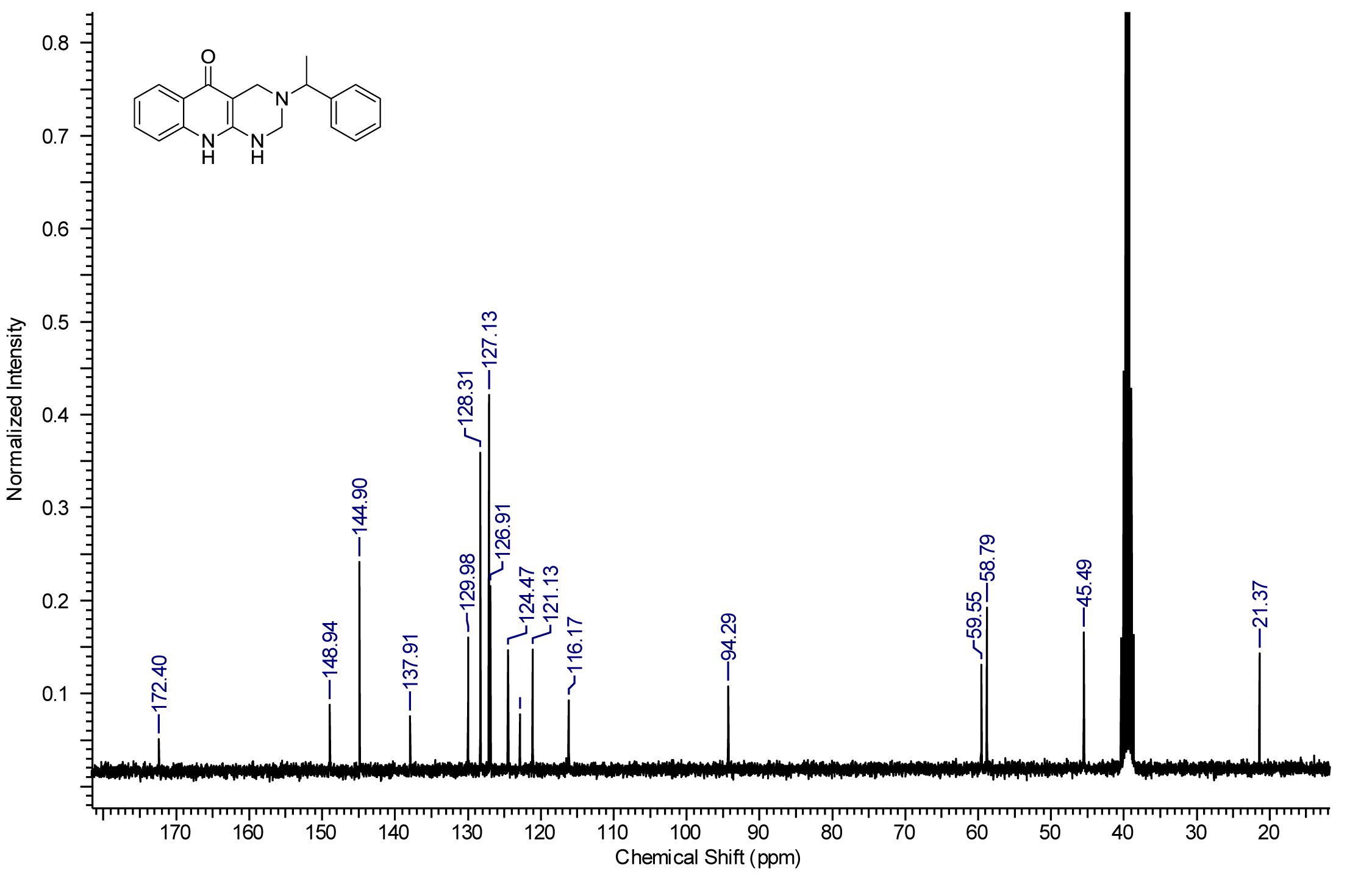

Supplement: S17 Fig — (TIF) [file pone.0175364.s017.tif]

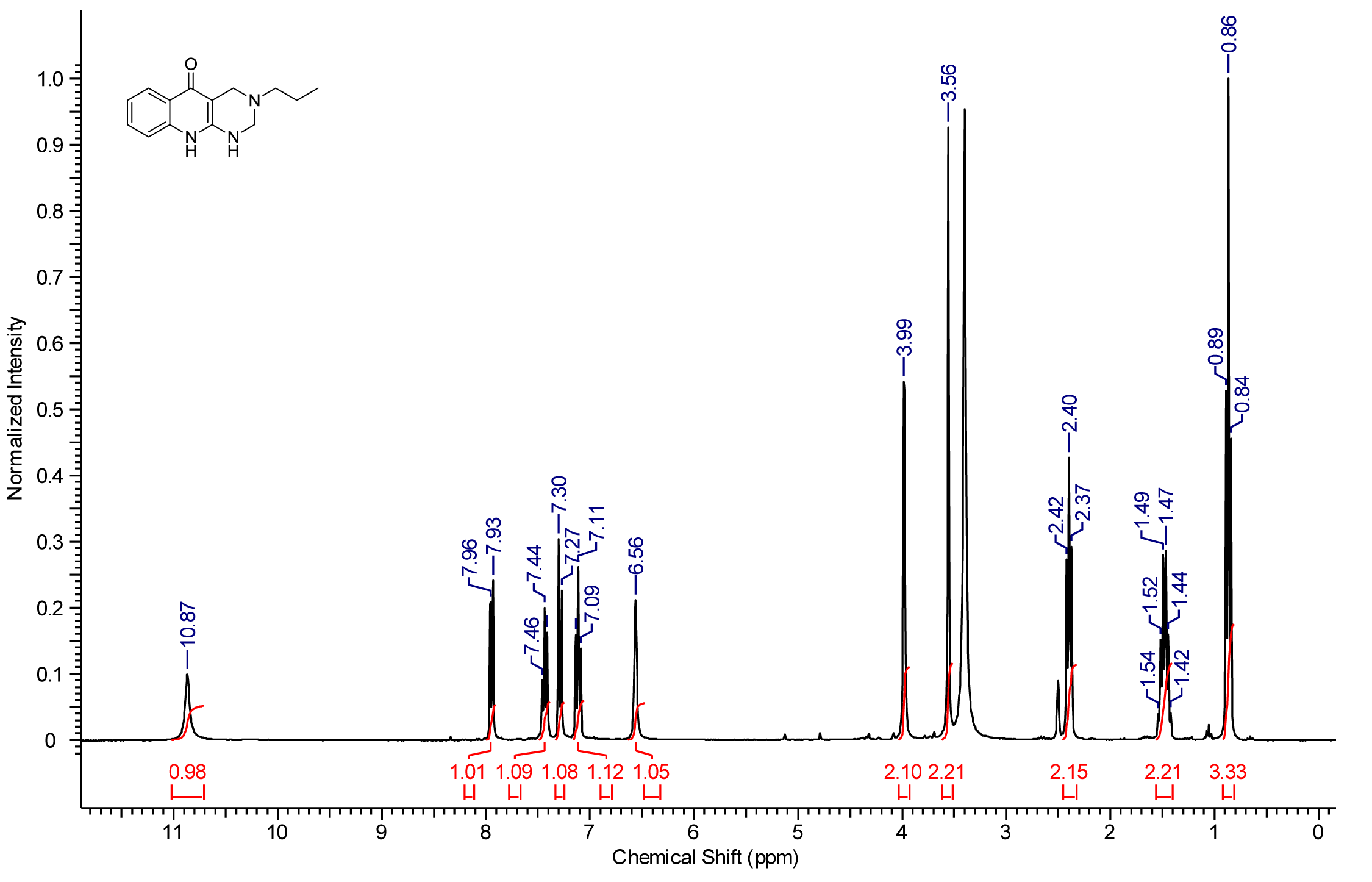

Supplement: S18 Fig — (TIF) [file pone.0175364.s018.tif]

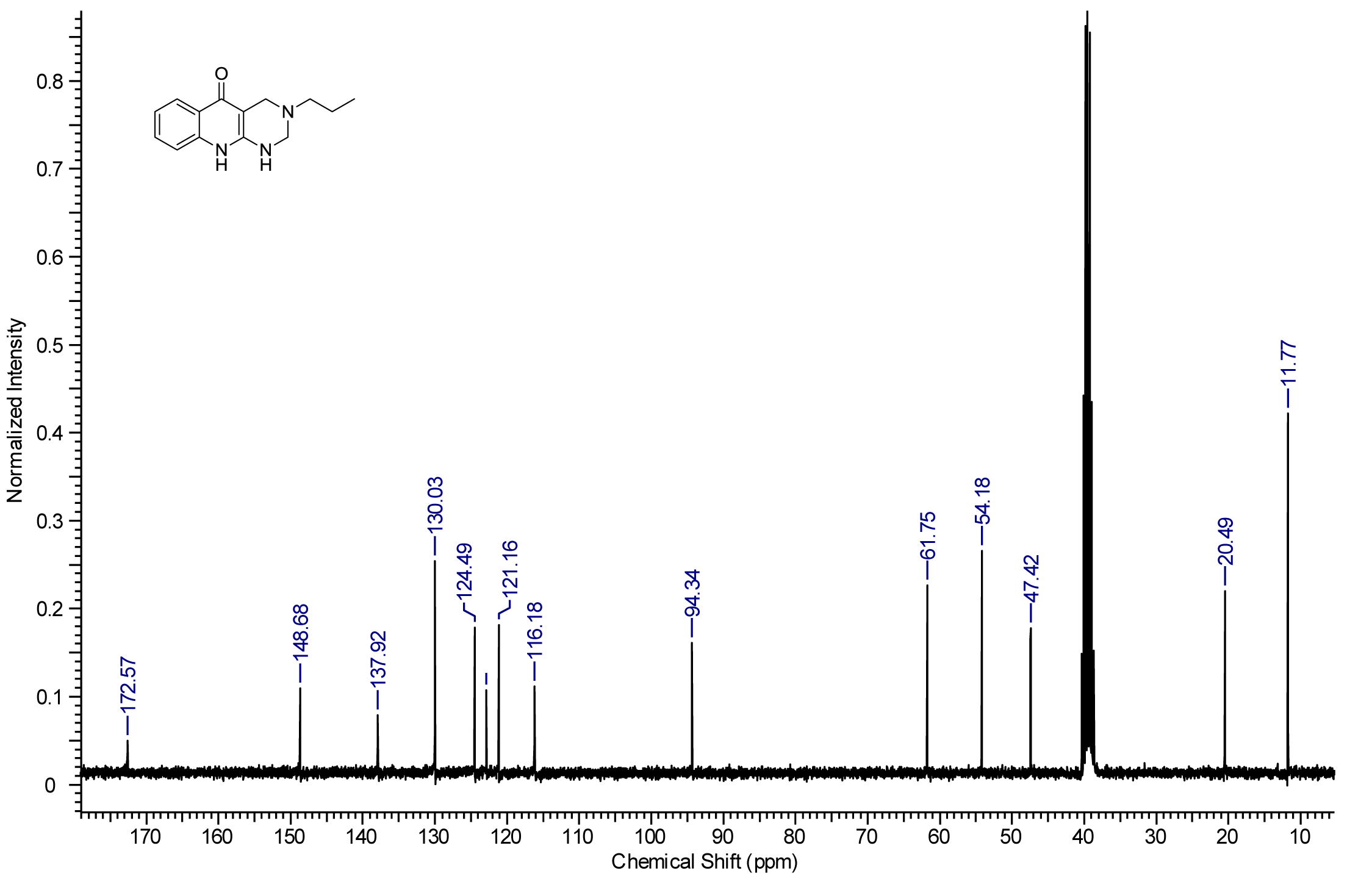

Supplement: S19 Fig — (TIF) [file pone.0175364.s019.tif]

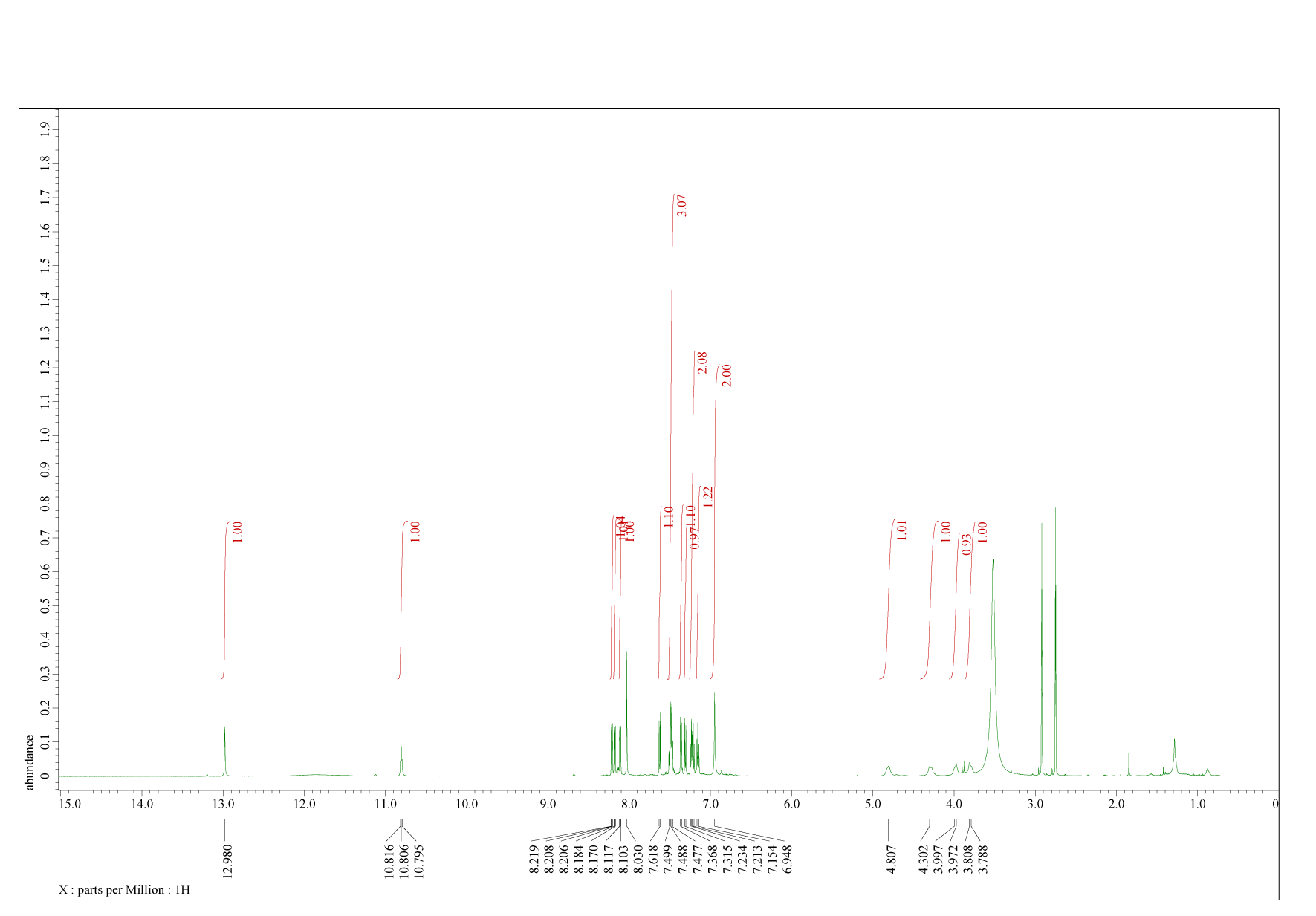

Supplement: S20 Fig — (TIF) [file pone.0175364.s020.tif]

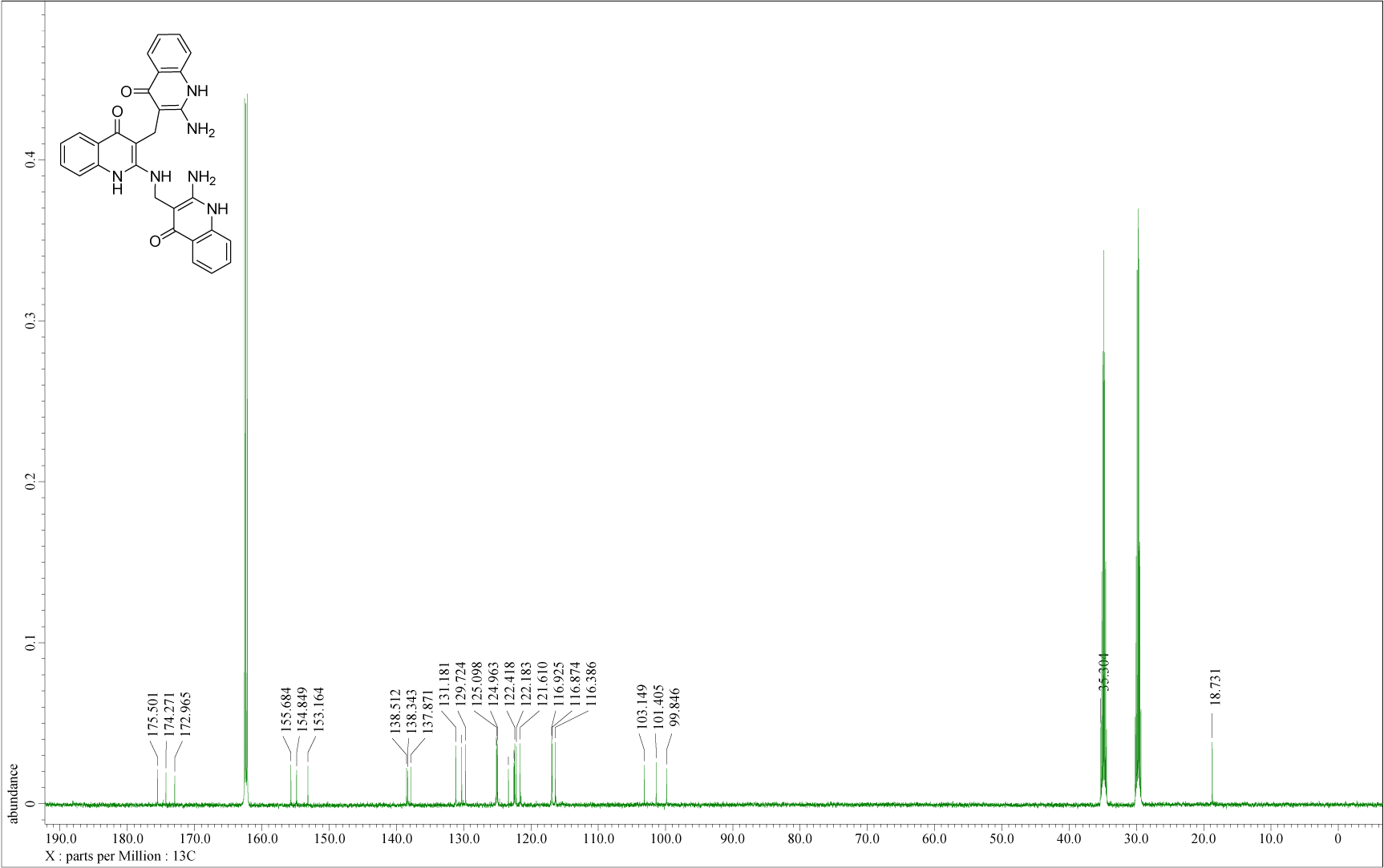

Supplement: S21 Fig — (TIF) [file pone.0175364.s021.tif]

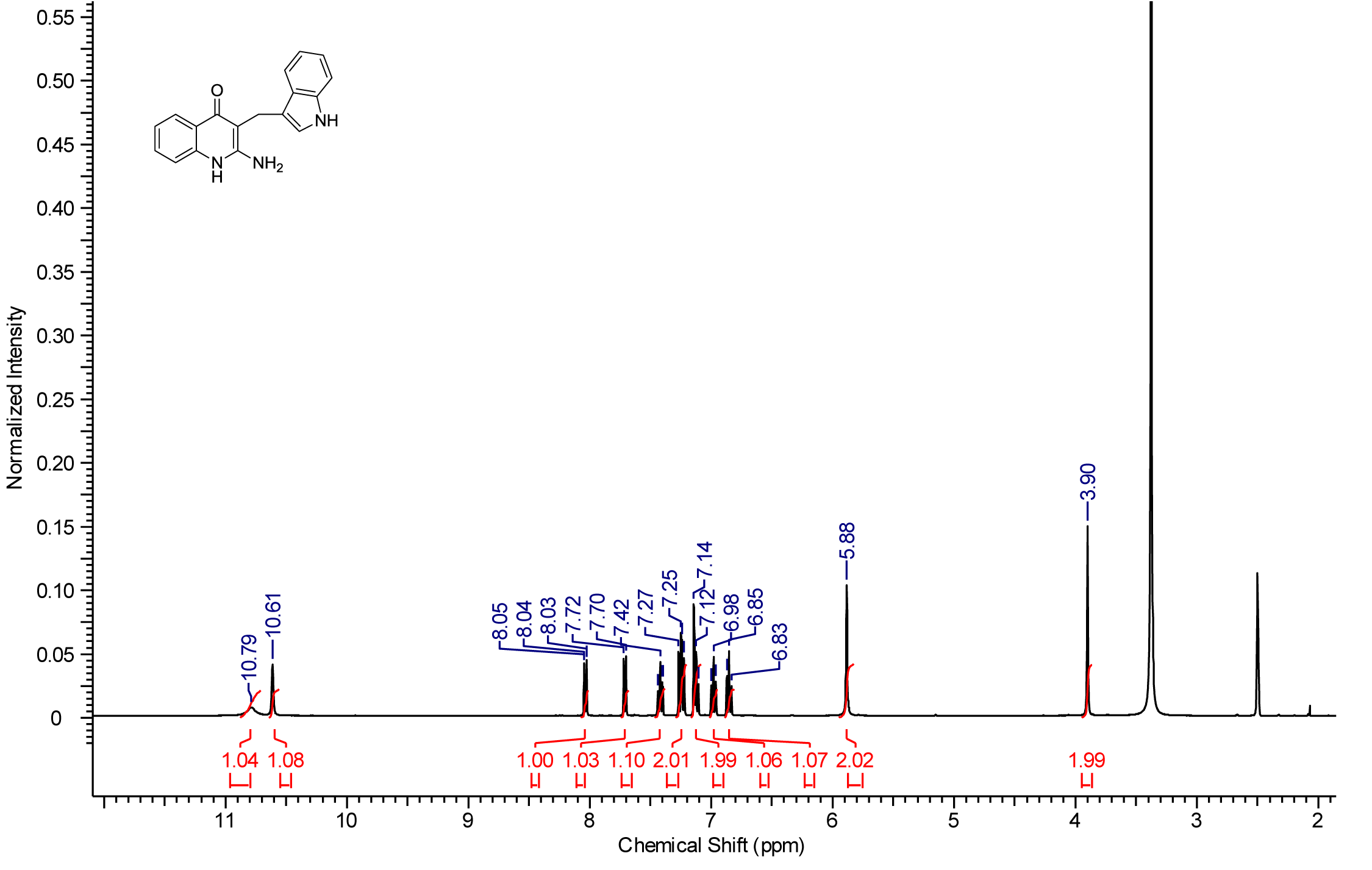

Supplement: S22 Fig — (TIF) [file pone.0175364.s022.tif]

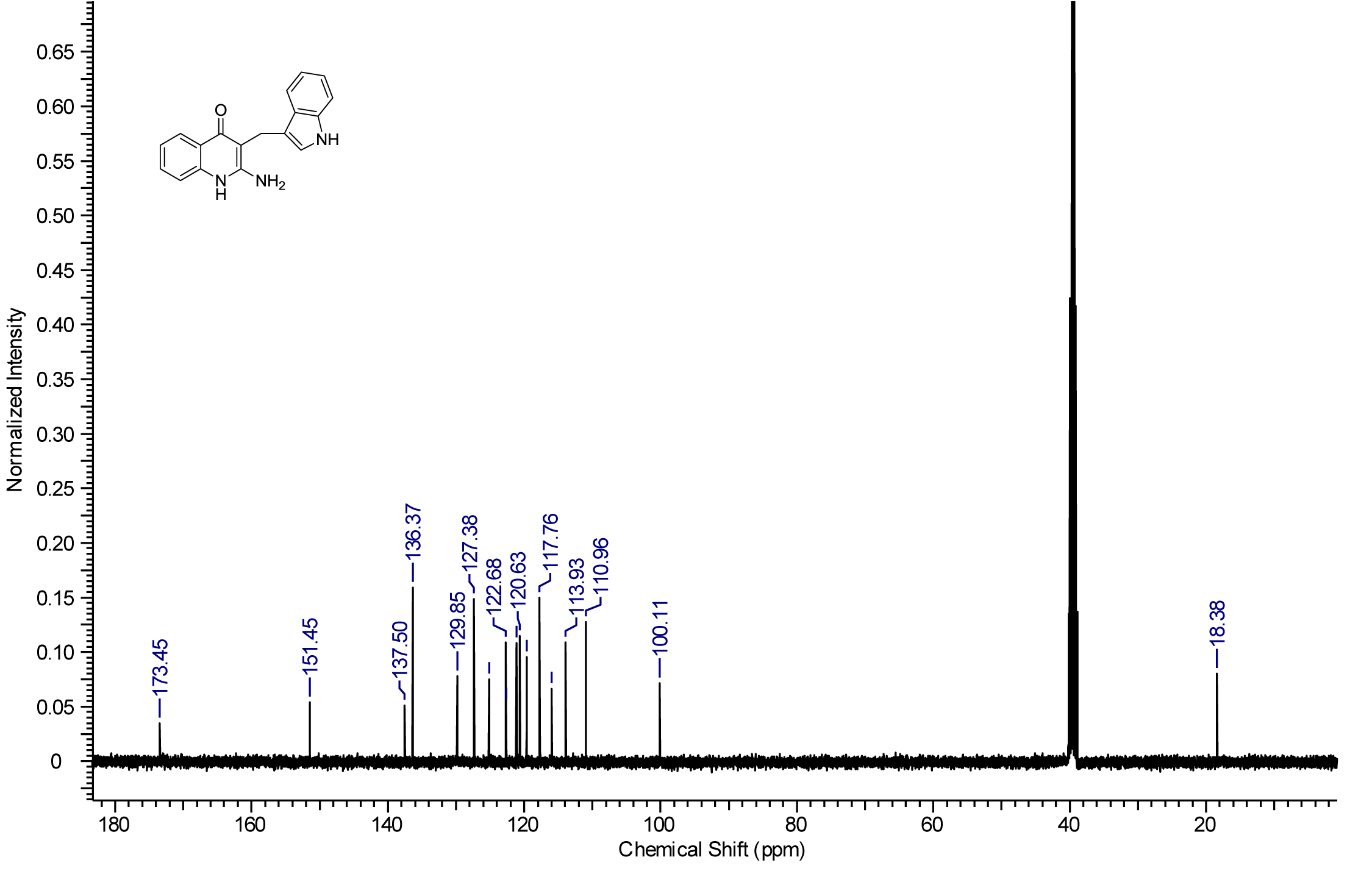

Supplement: S23 Fig — (TIF) [file pone.0175364.s023.tif]

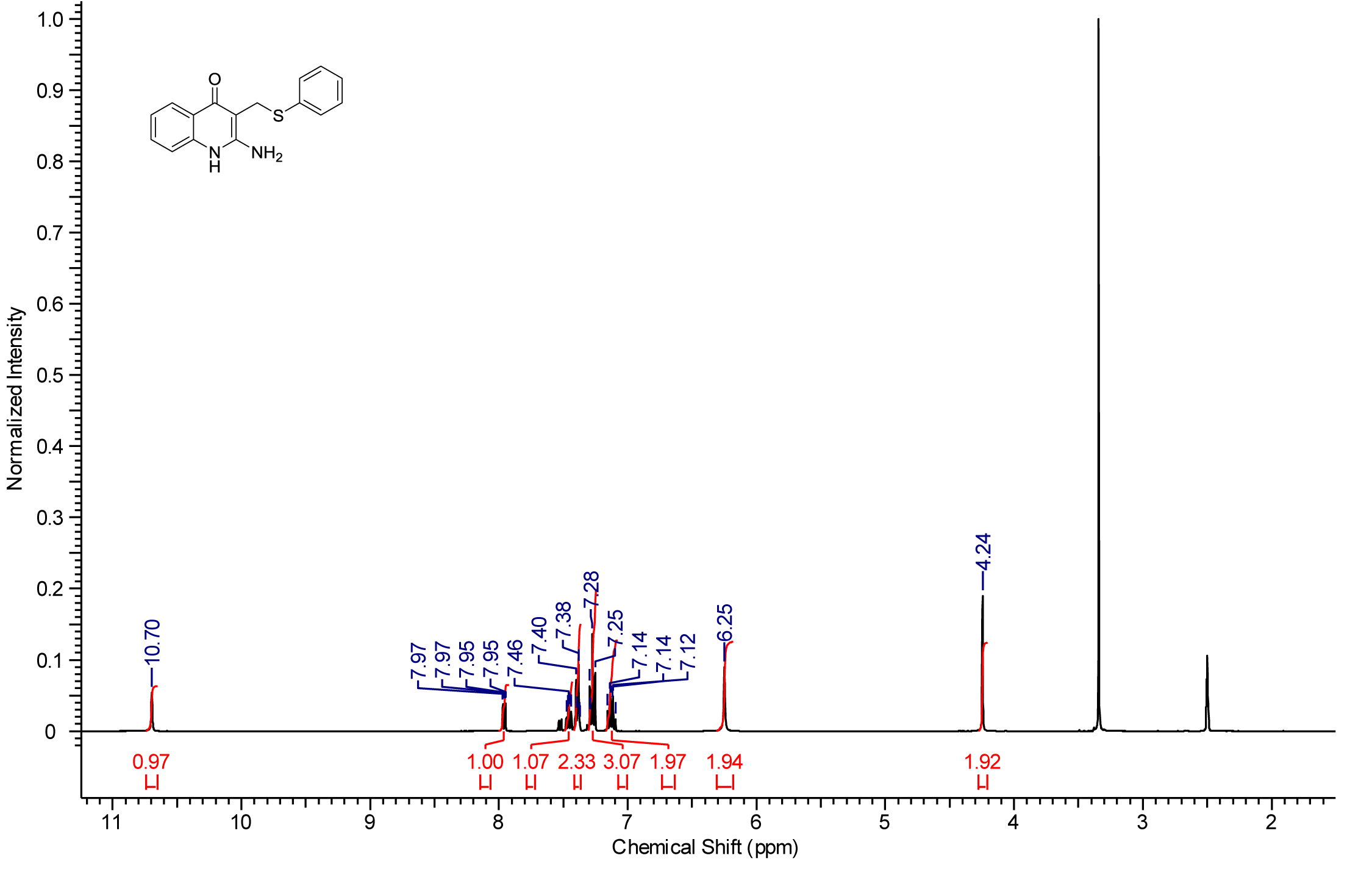

Supplement: S24 Fig — (TIF) [file pone.0175364.s024.tif]

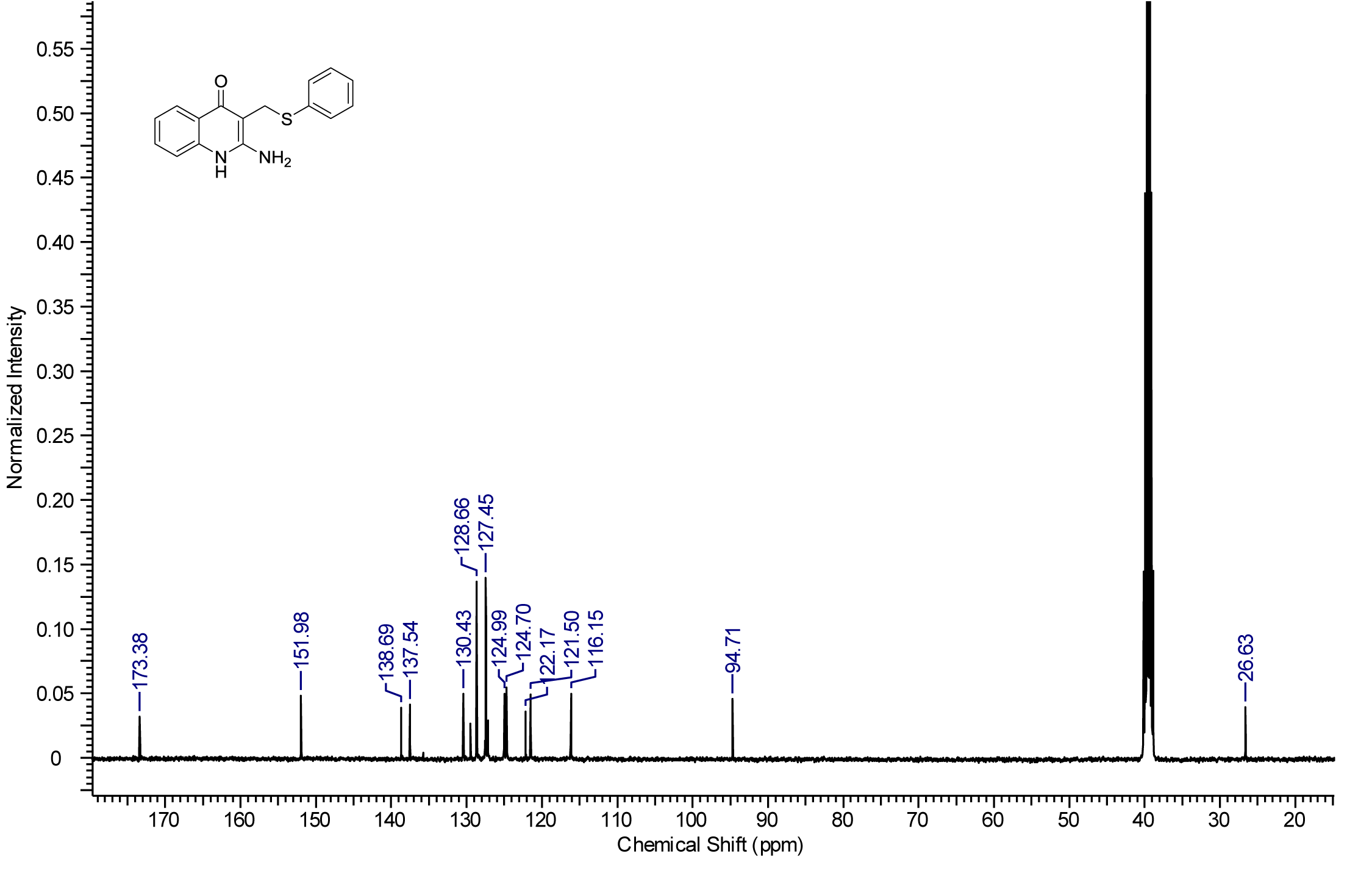

Supplement: S25 Fig — (TIF) [file pone.0175364.s025.tif]

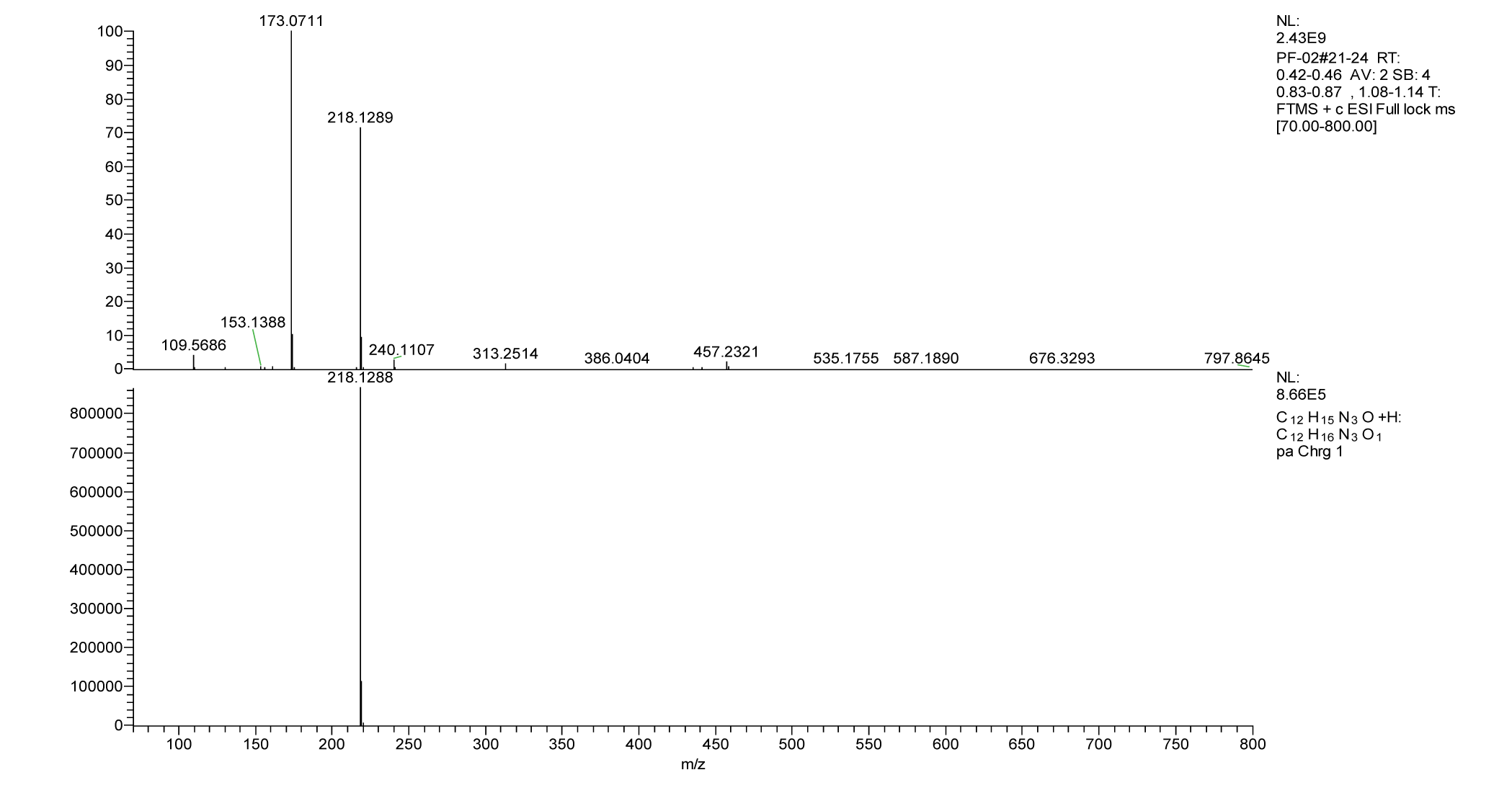

Supplement: S26 Fig — (TIF) [file pone.0175364.s026.tif]

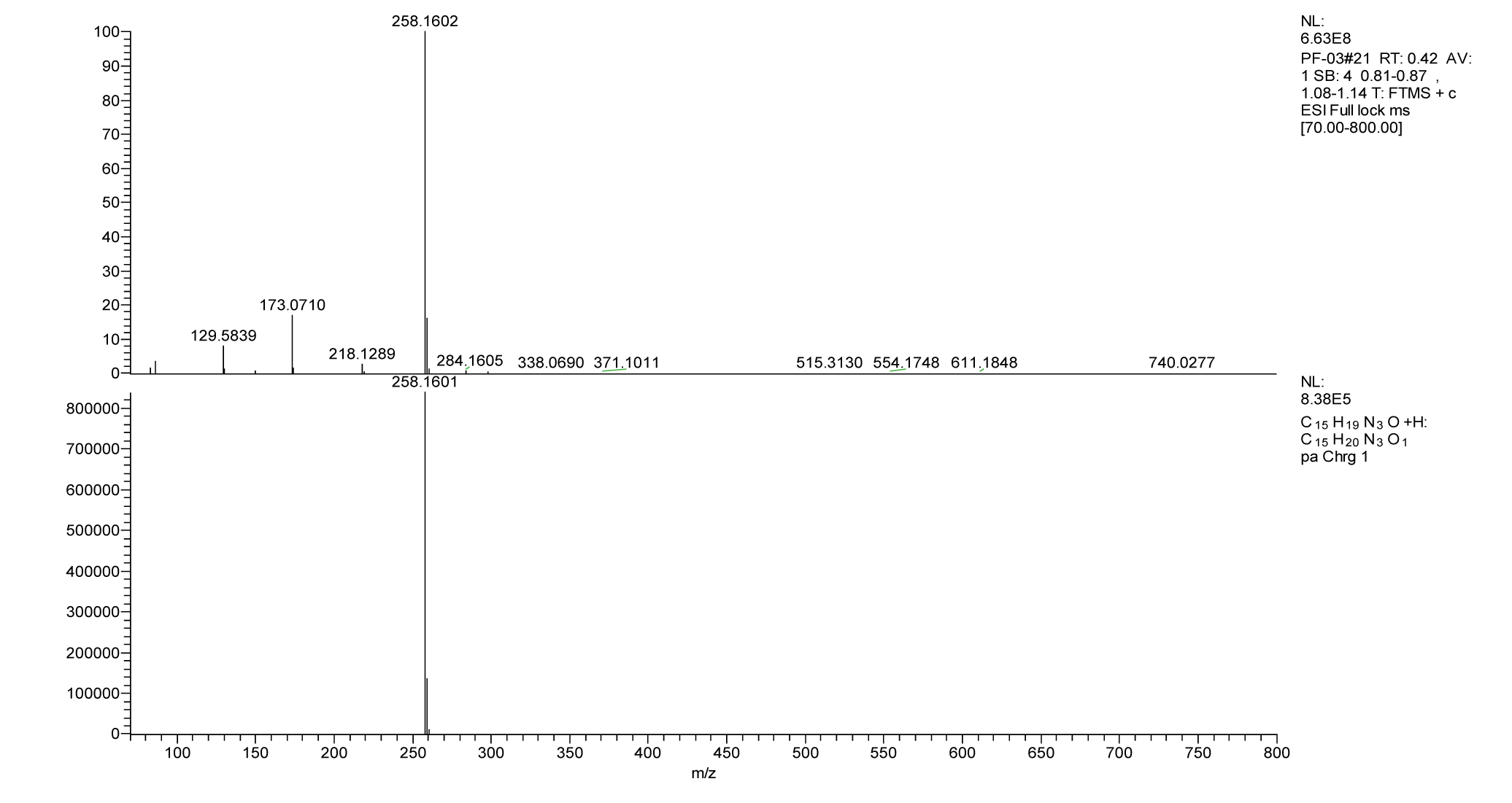

Supplement: S27 Fig — (TIF) [file pone.0175364.s027.tif]

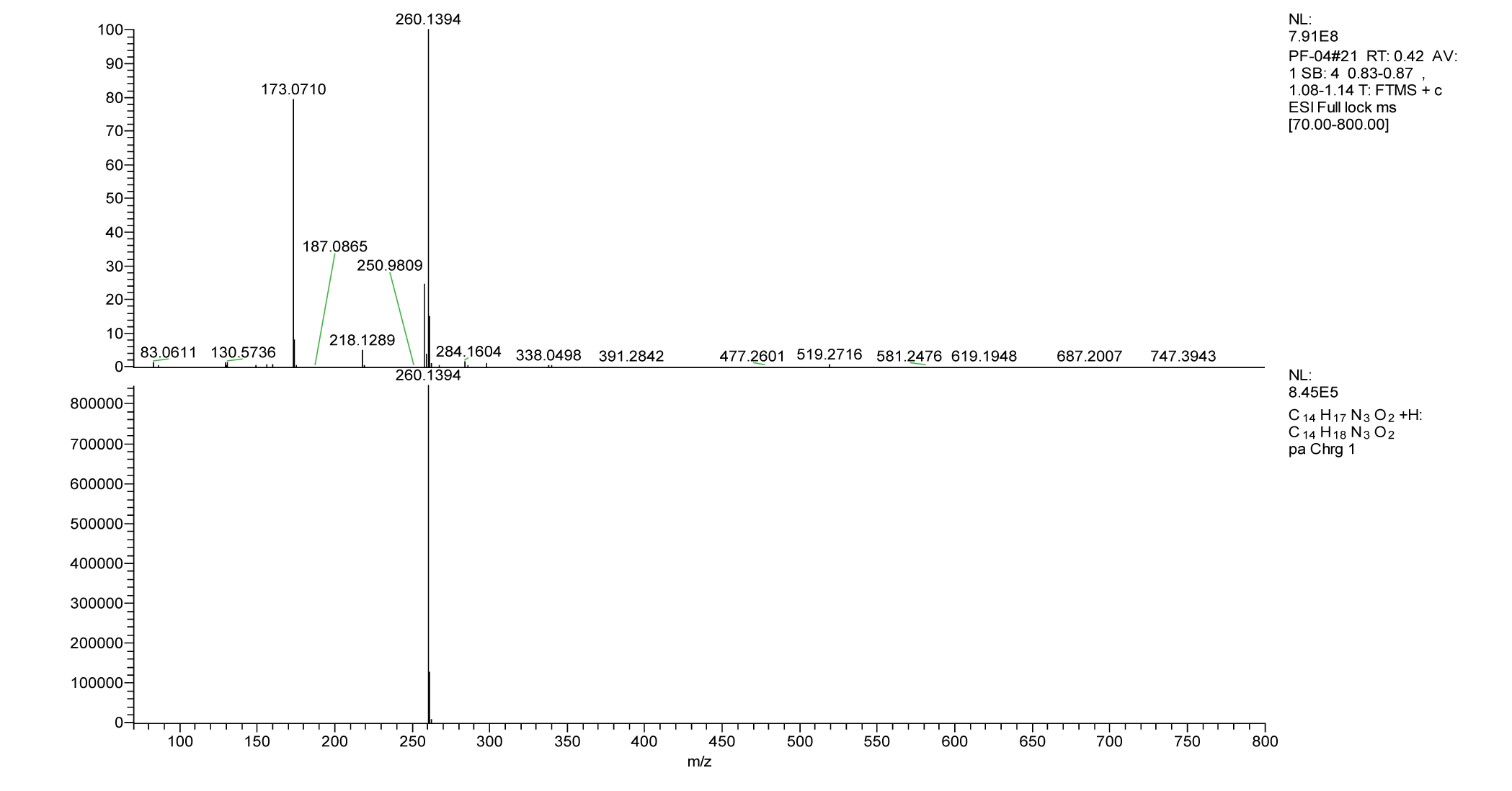

Supplement: S28 Fig — (TIF) [file pone.0175364.s028.tif]

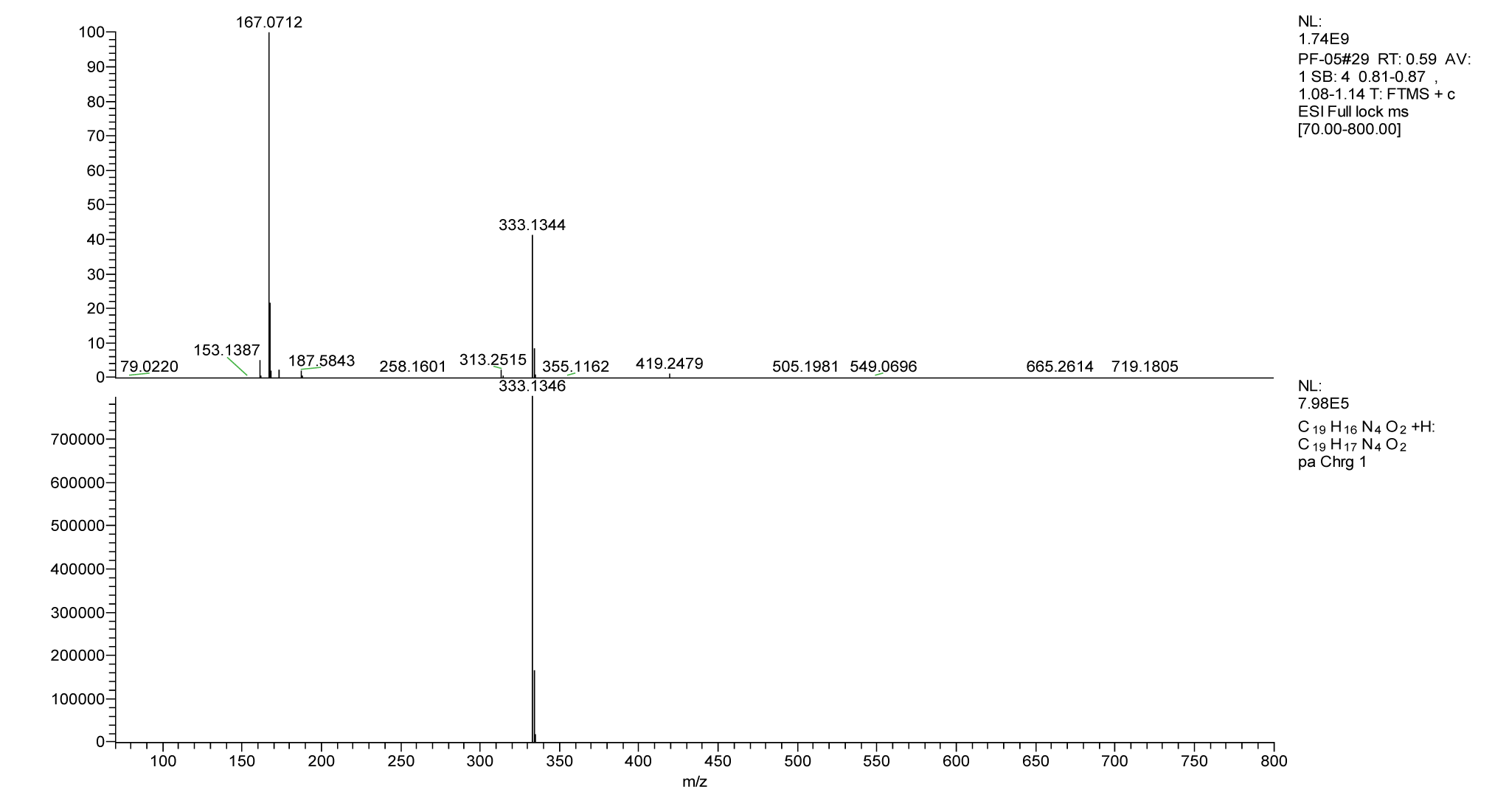

Supplement: S29 Fig — (TIF) [file pone.0175364.s029.tif]

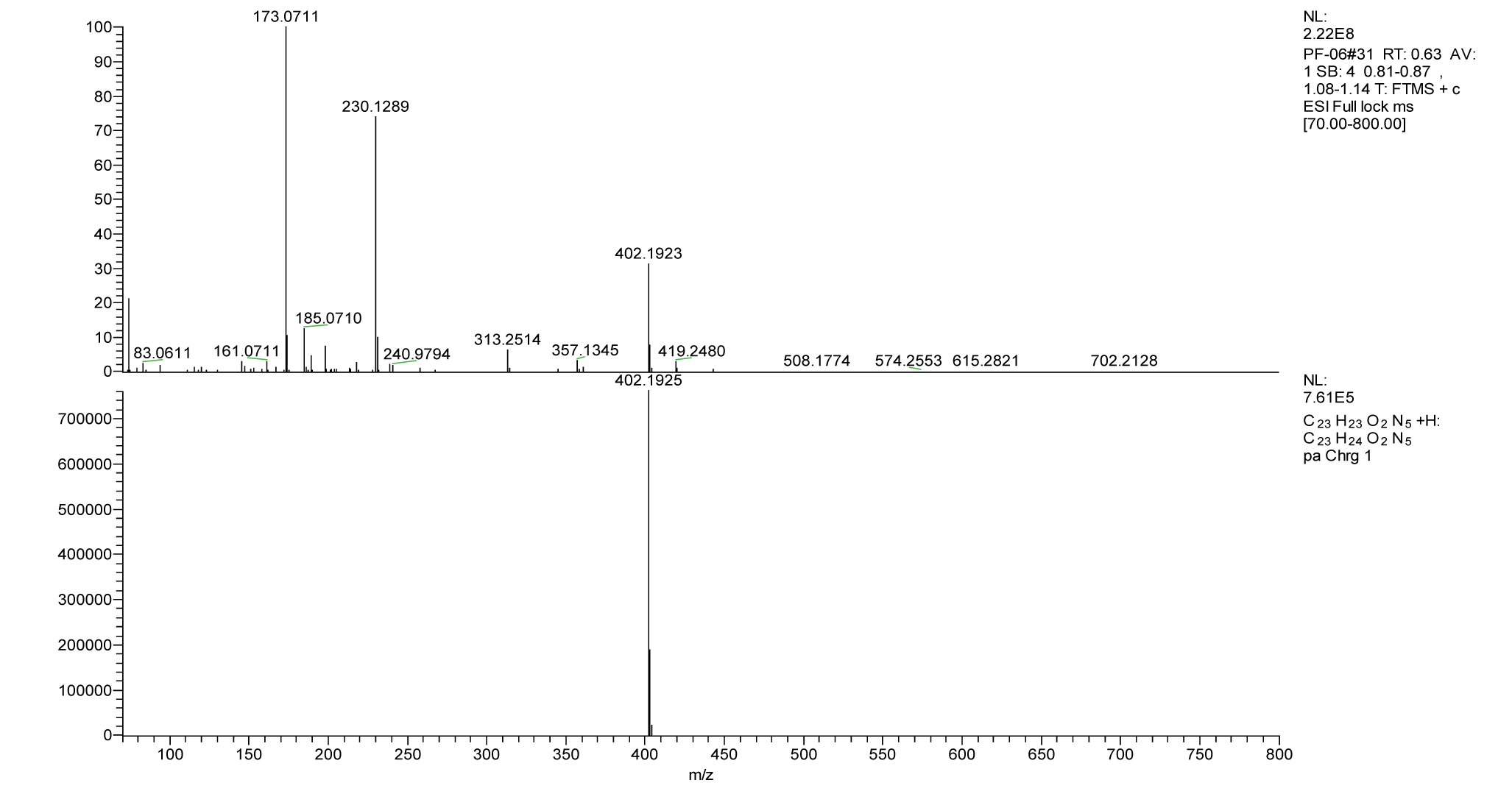

Supplement: S30 Fig — (TIF) [file pone.0175364.s030.tif]

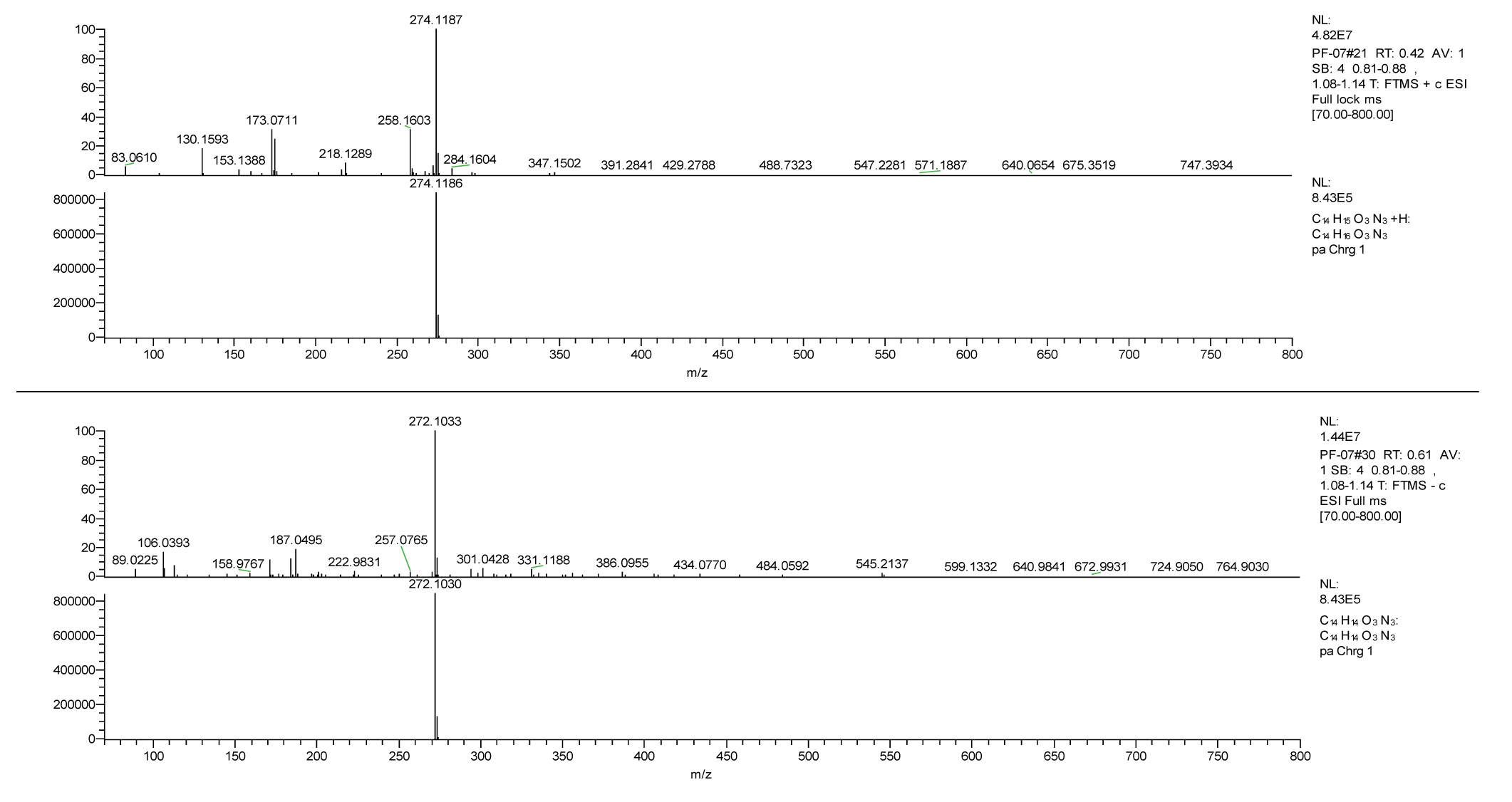

Supplement: S31 Fig — (TIF) [file pone.0175364.s031.tif]

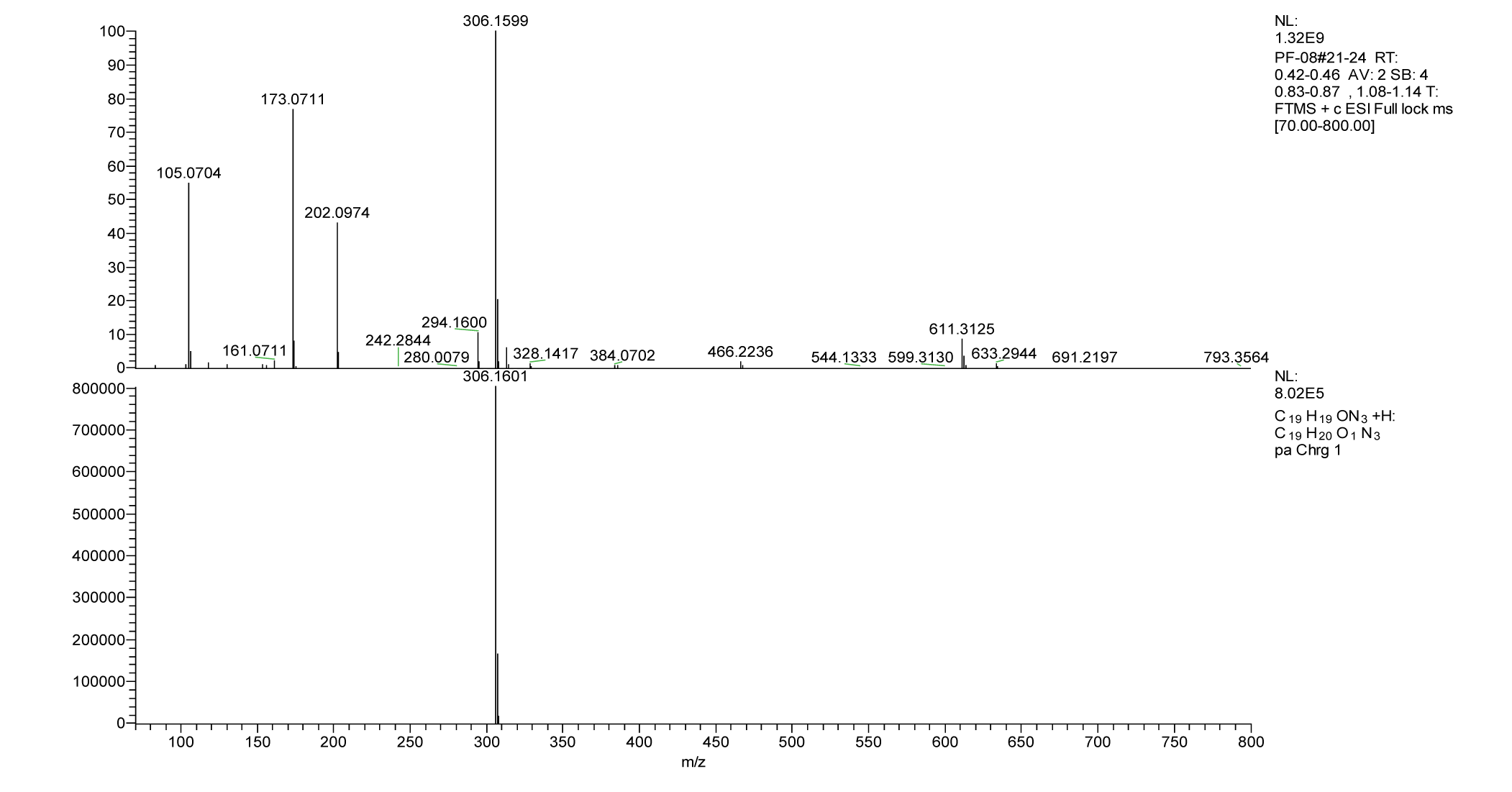

Supplement: S32 Fig — (TIF) [file pone.0175364.s032.tif]

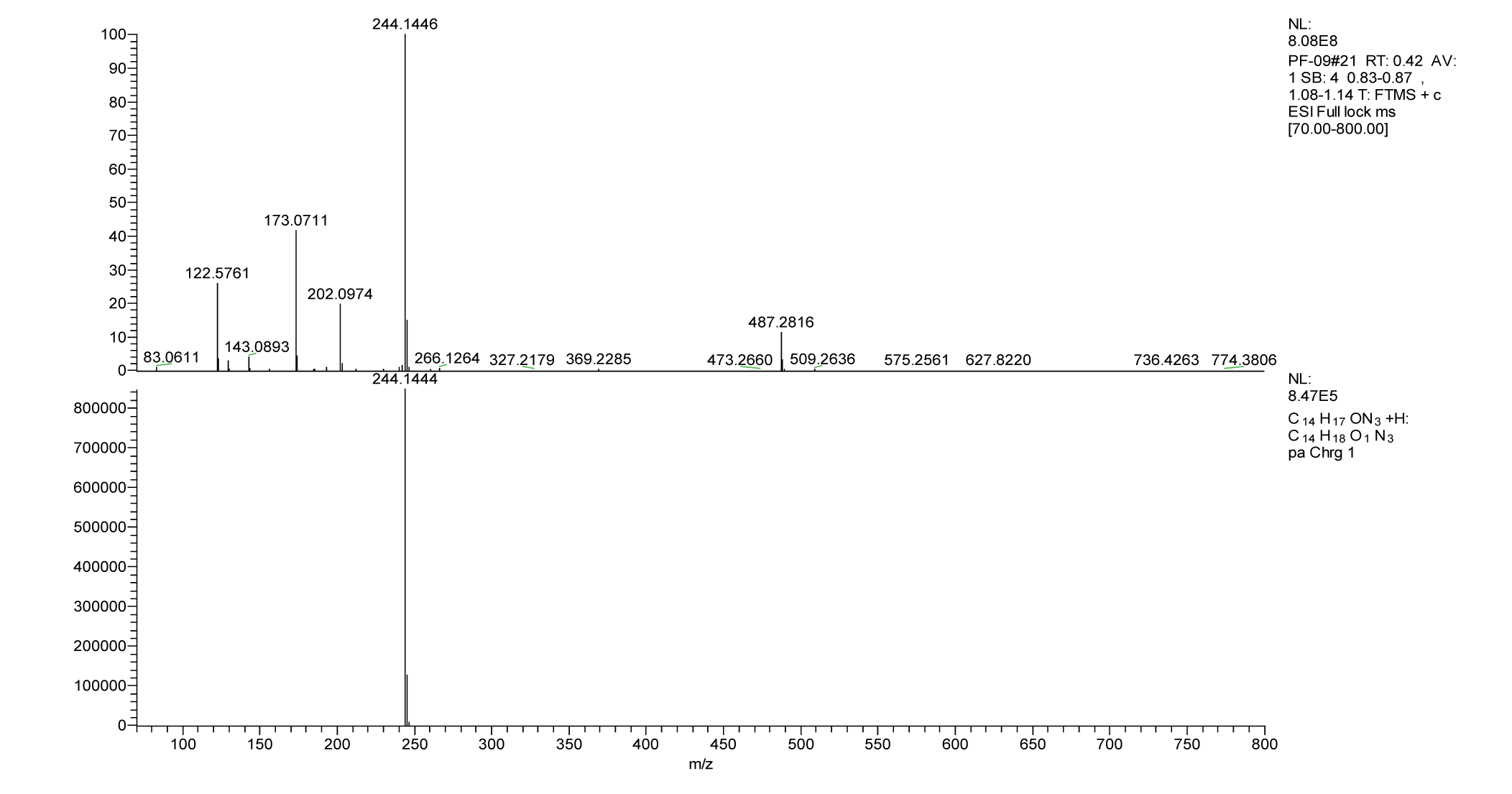

Supplement: S33 Fig — (TIF) [file pone.0175364.s033.tif]

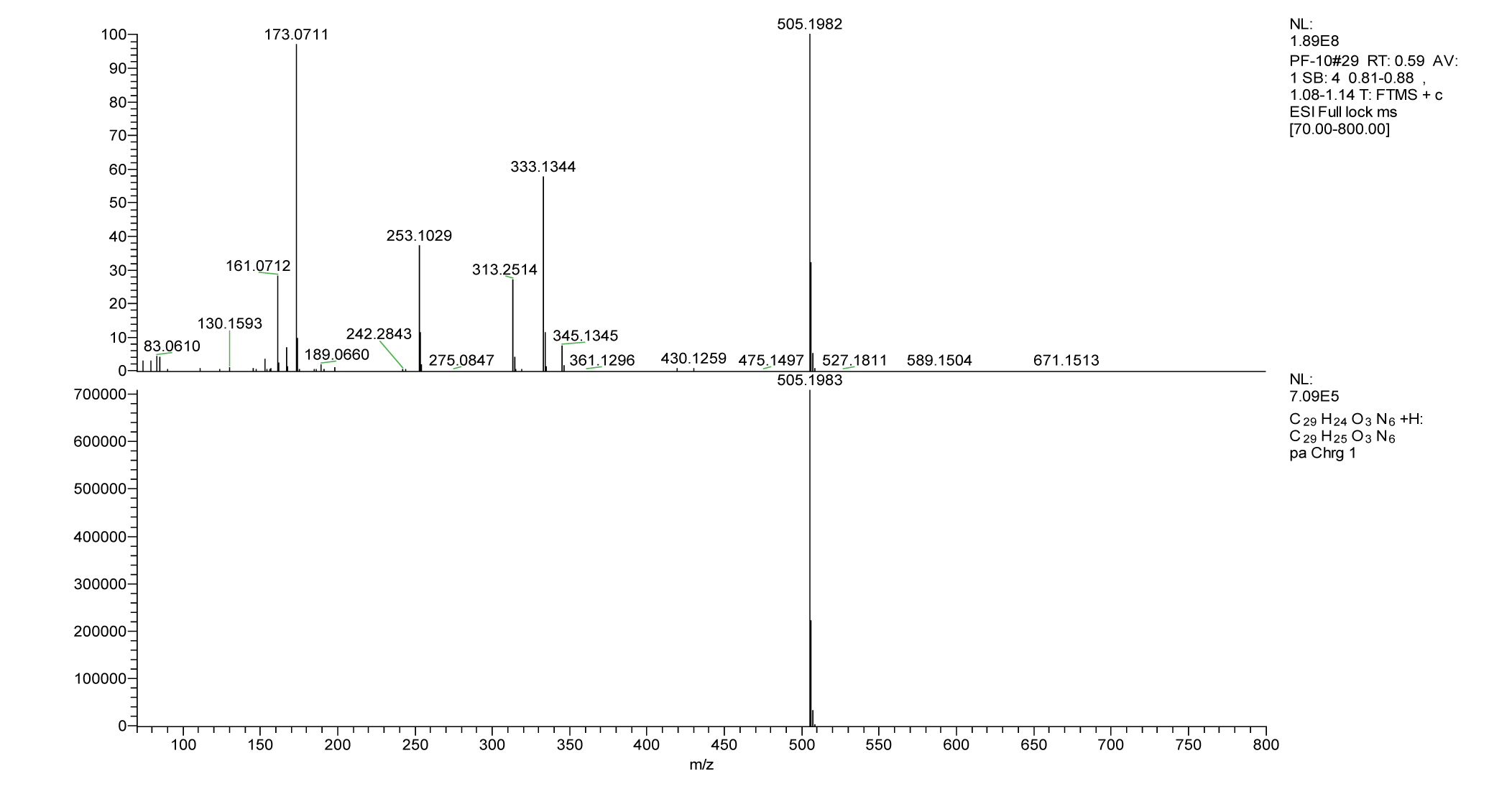

Supplement: S34 Fig — (TIF) [file pone.0175364.s034.tif]

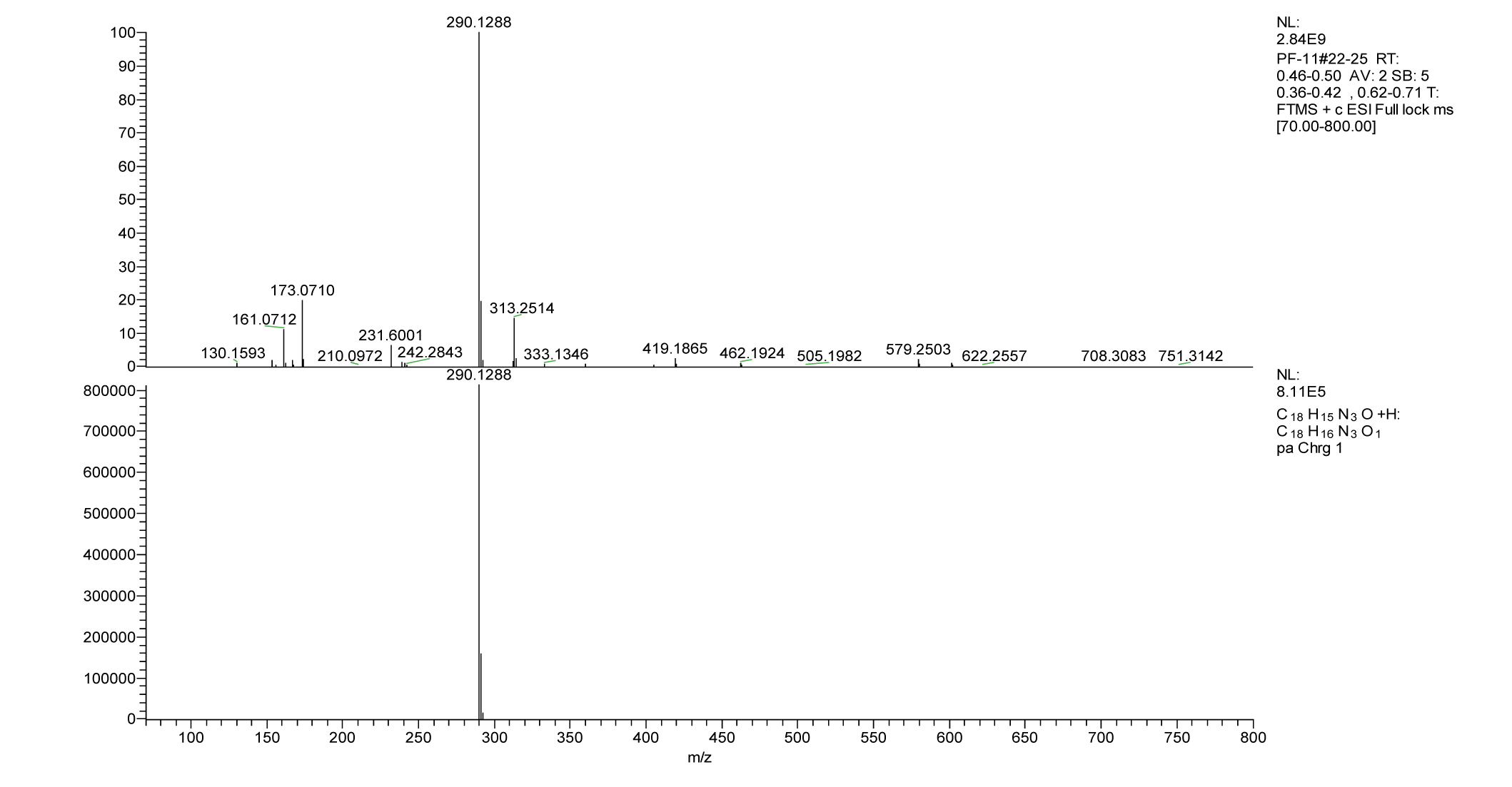

Supplement: S35 Fig — (TIF) [file pone.0175364.s035.tif]

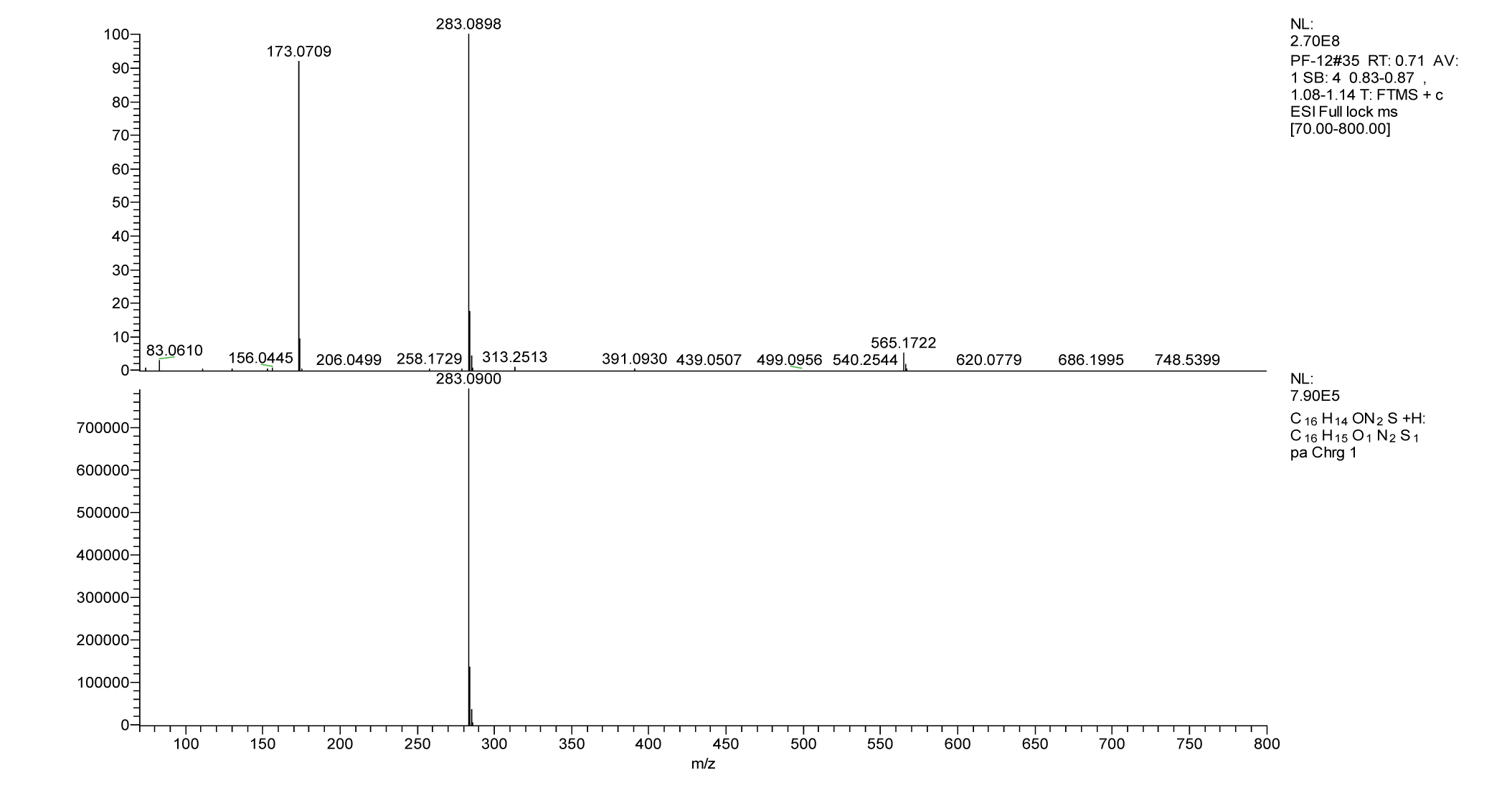

Supplement: S36 Fig — (TIF) [file pone.0175364.s036.tif]

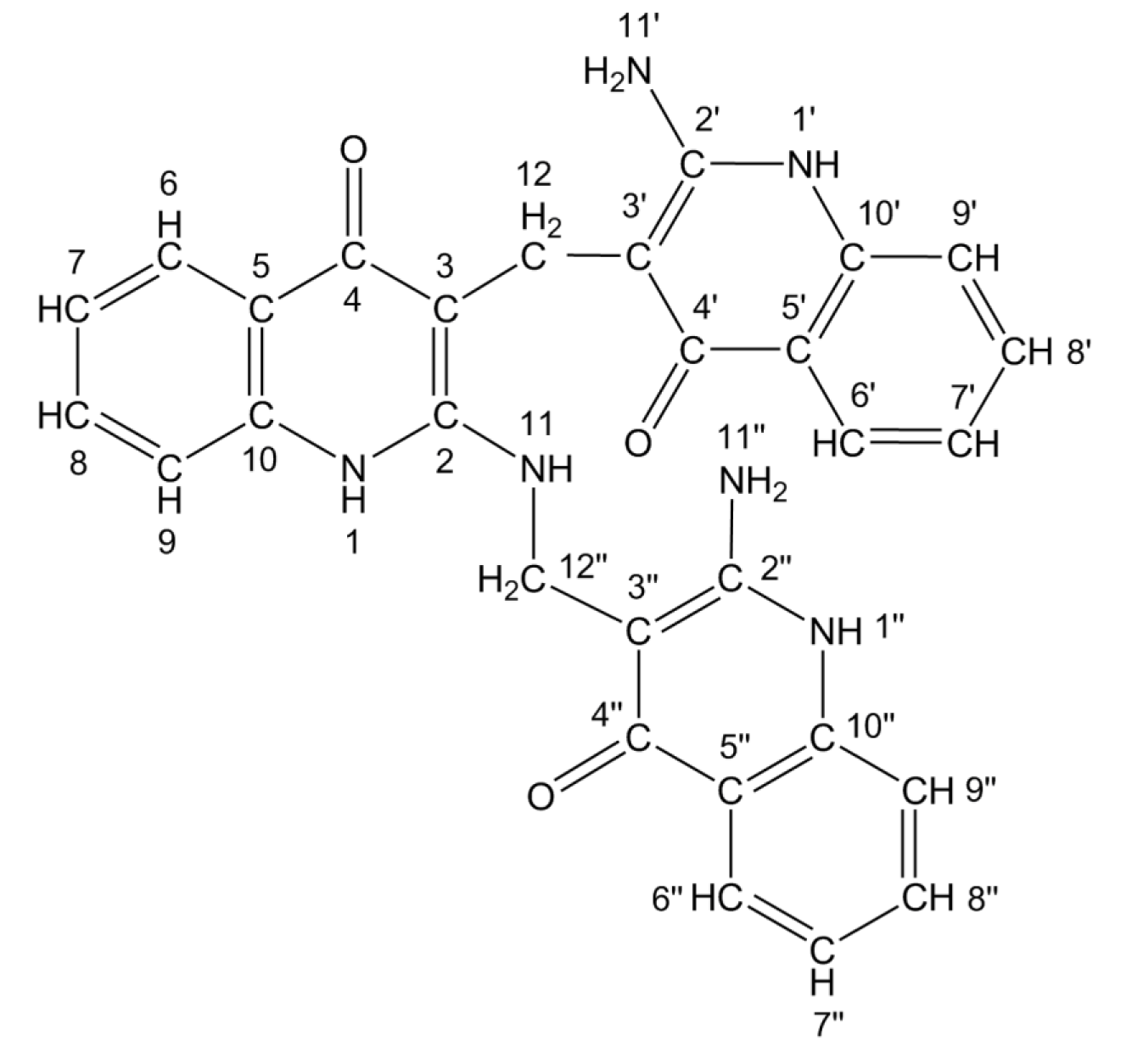

Supplement: S37 Fig — (TIF) [file pone.0175364.s037.tif]

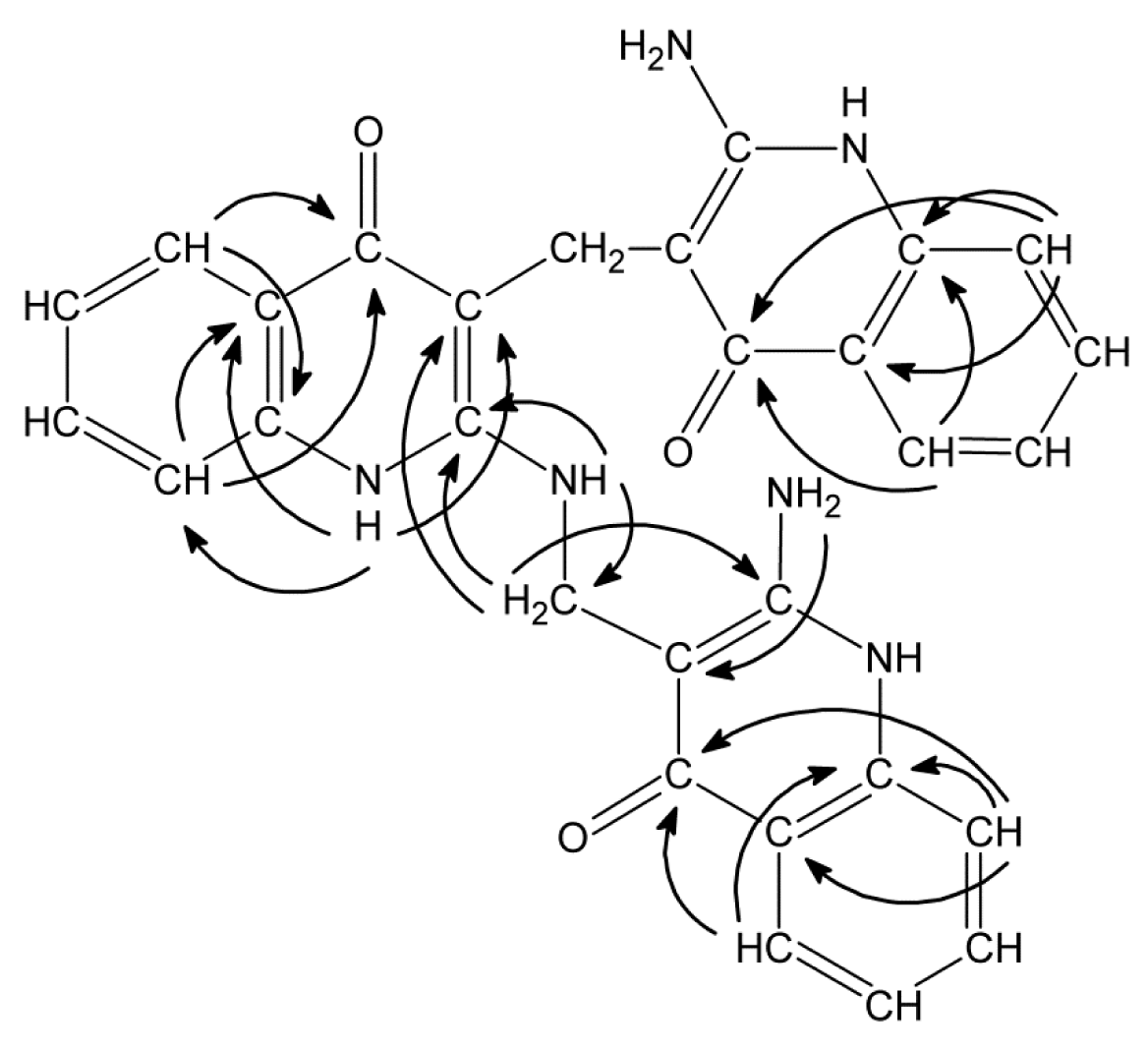

Supplement: S38 Fig — All correlations of protons H-7, H-8, H-7’, H-8’, H-7” and H-8” are omitted for simplicity. Correlations of protons H-6, H-9, H-6’, H-9’, H-6” and H-9” to protonated-carbons of the same ring are also omitted for clarity. (TIF) [file pone.0175364.s038.tif]

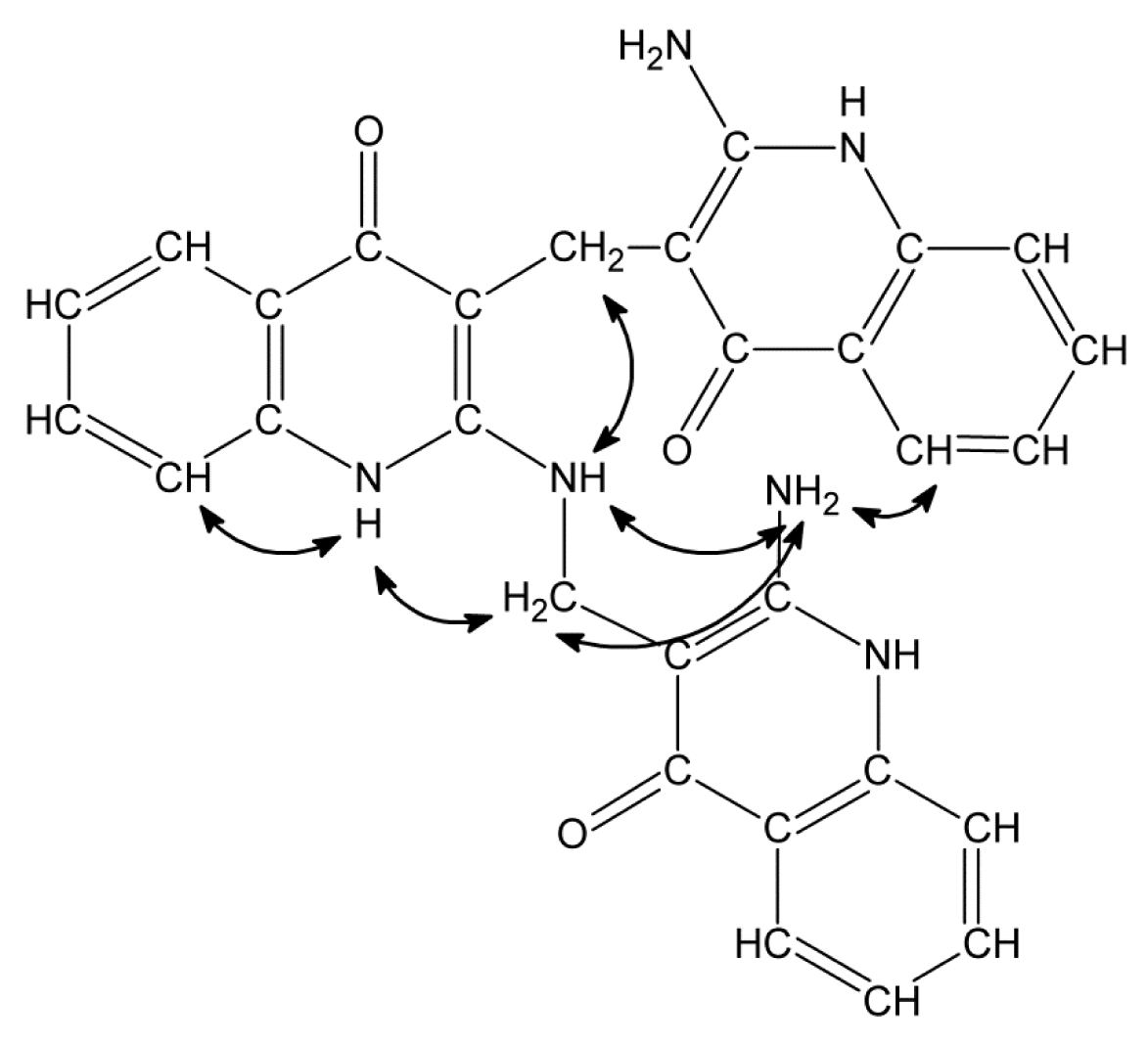

Supplement: S39 Fig — (TIF) [file pone.0175364.s039.tif]

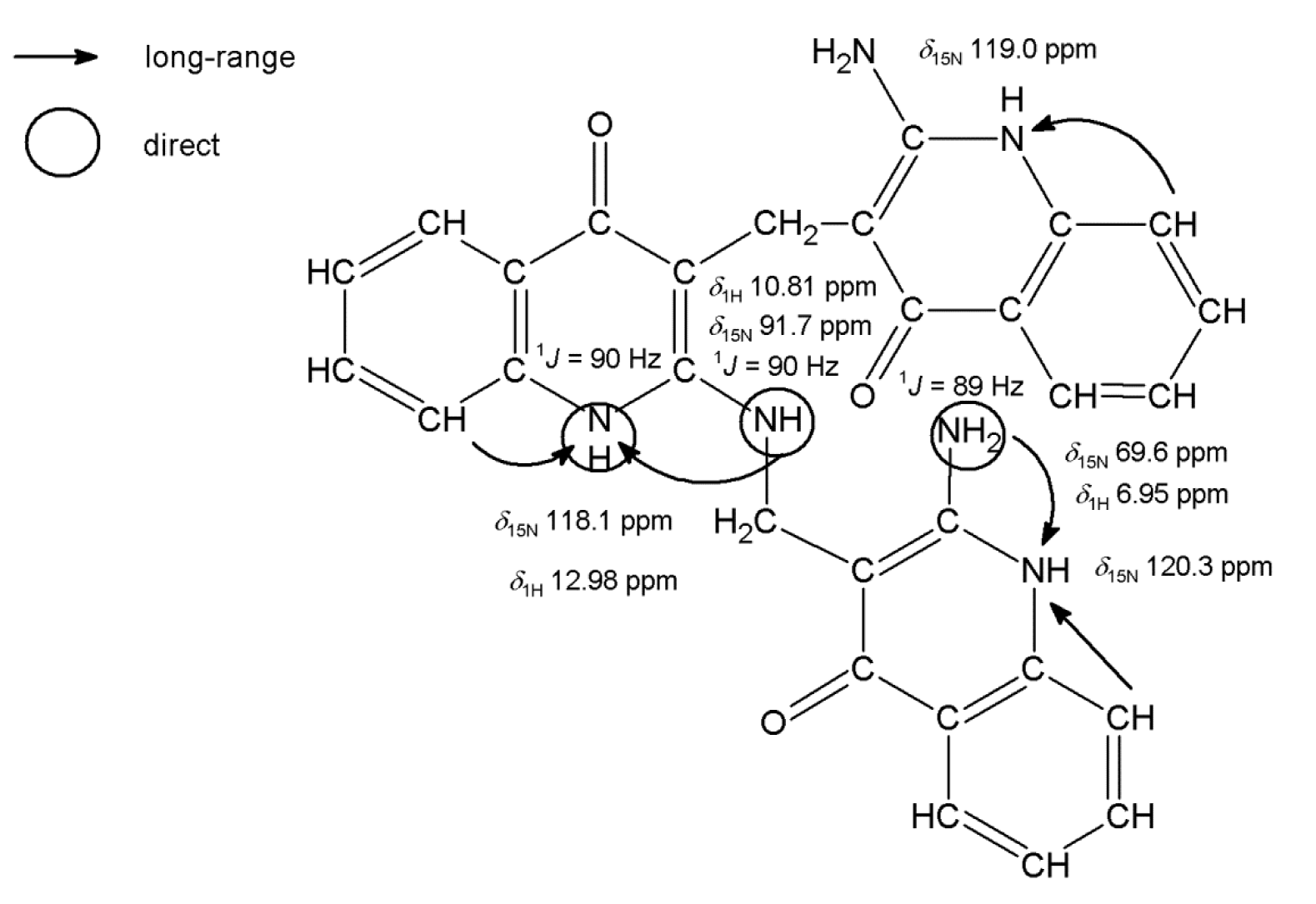

Supplement: S40 Fig — (TIF) [file pone.0175364.s040.tif]

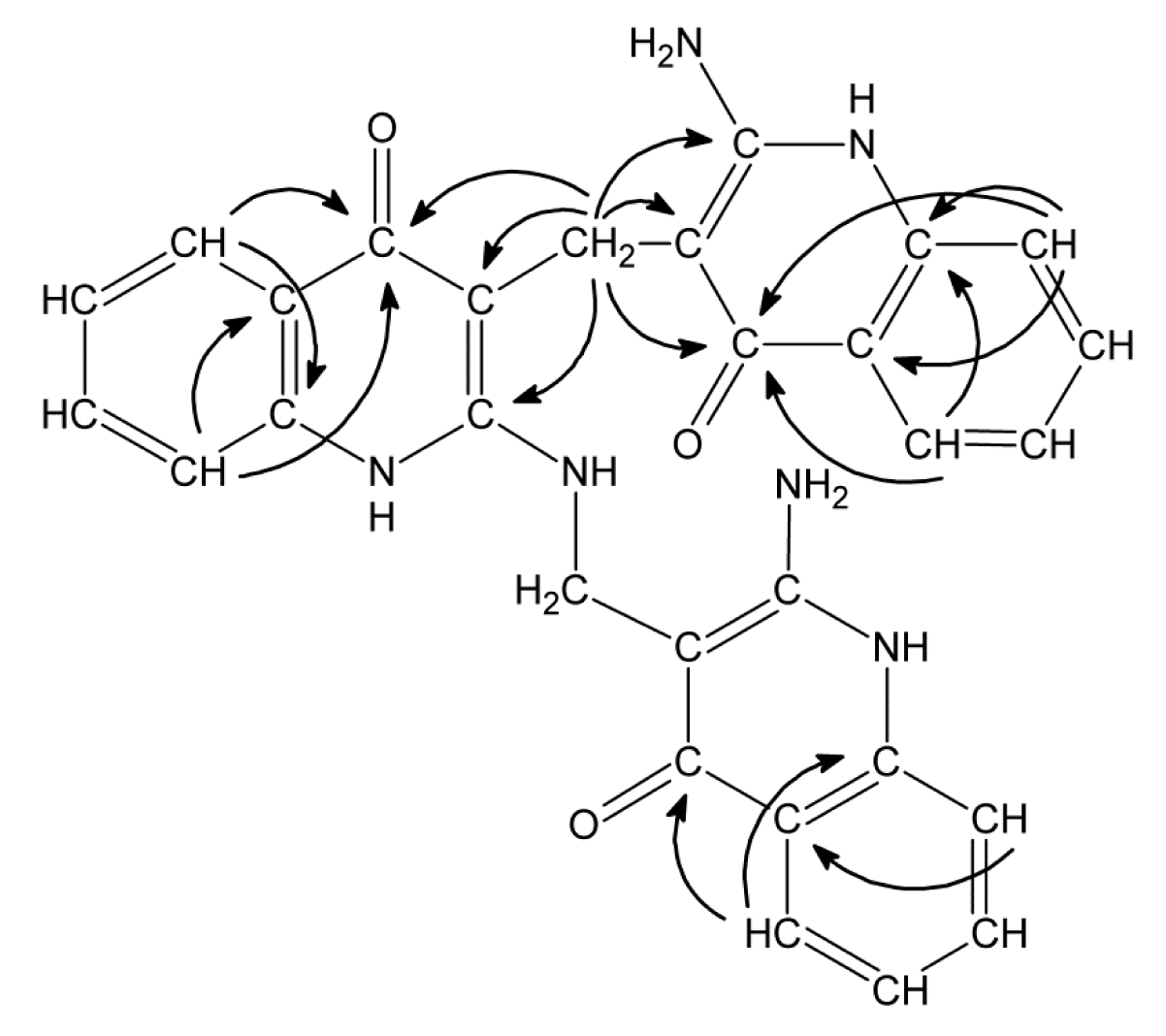

Supplement: S41 Fig — All correlations of protons H-7, H-8, H-7’, H-8’, H-7” and H-8” are omitted for simplicity. Correlations of protons H-6, H-9, H-6’, H-9’, H-6” and H-9” to protonated-carbons of the same ring are also omitted for clarity. (TIF) [file pone.0175364.s041.tif]

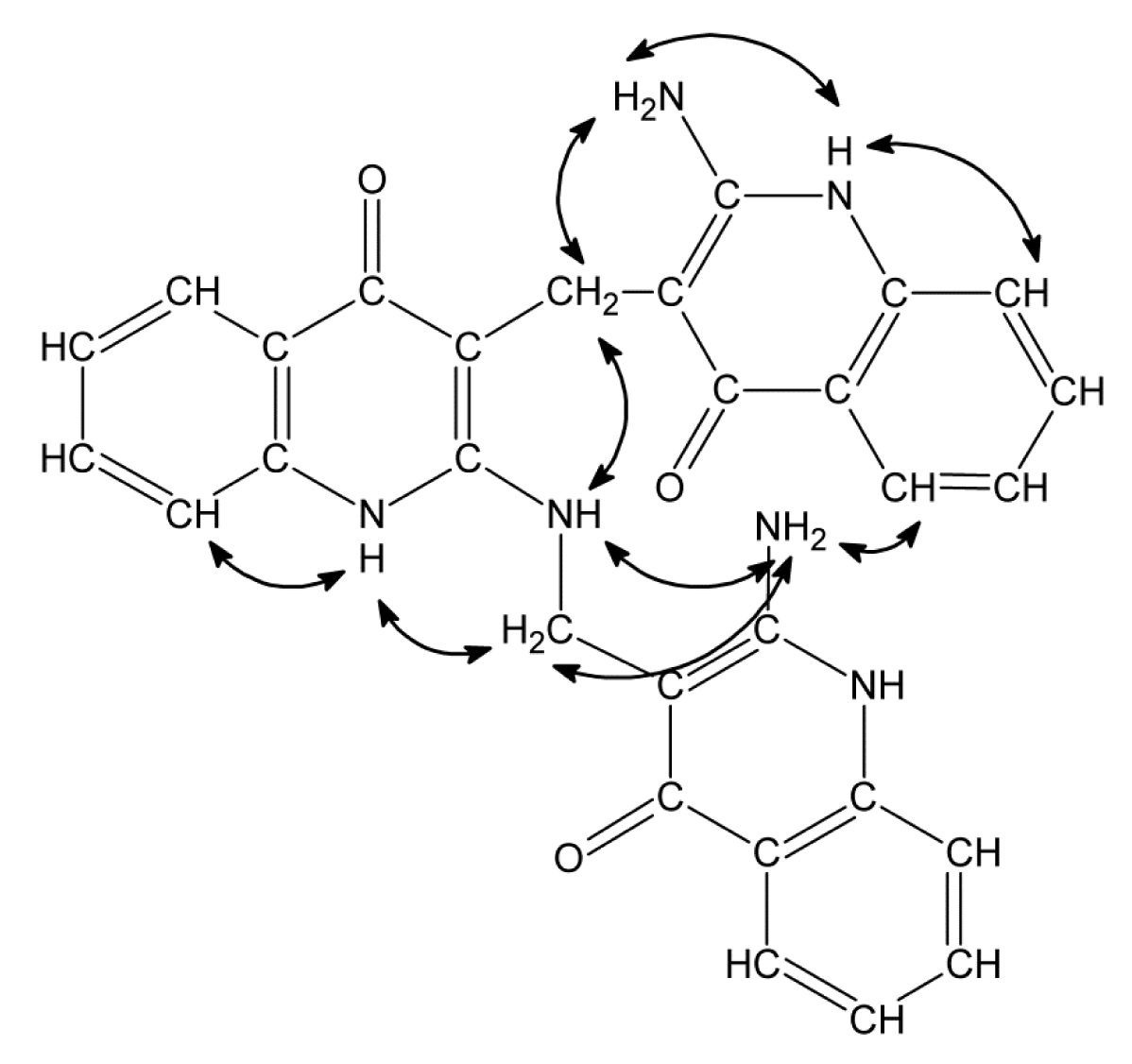

Supplement: S42 Fig — (TIF) [file pone.0175364.s042.tif]

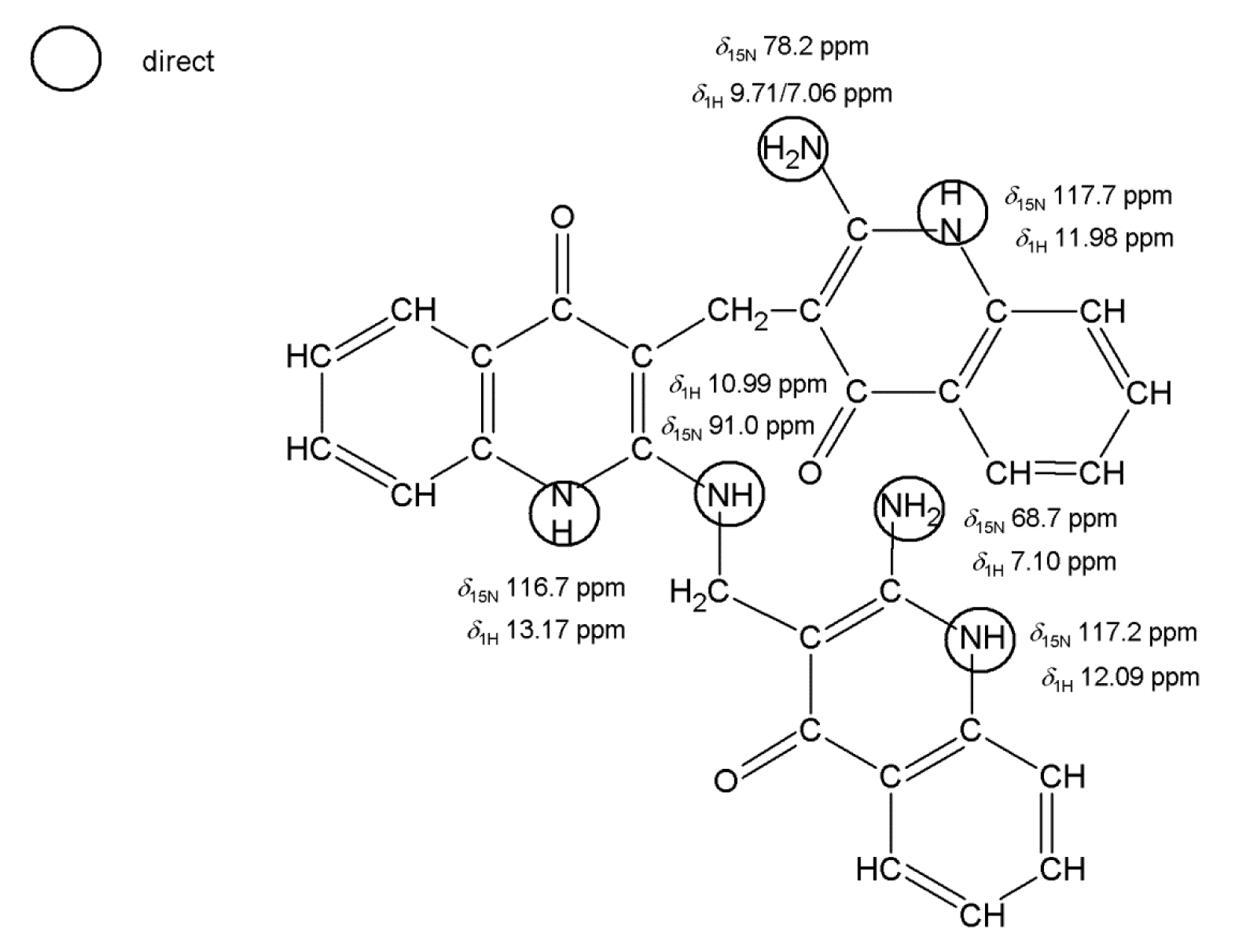

Supplement: S43 Fig — (TIF) [file pone.0175364.s043.tif]
