# Supplementary material for: Study of 2-aminoquinolin-4(1H)-one under Mannich and retro-Mannich reaction
Source: PLoS One. 2017 May 30;12(5):e0175364. doi: 10.1371/journal.pone.0175364 (PMC5448738; doi:10.1371/journal.pone.0175364)
Supplement: S1 File — (DOCX) [file pone.0175364.s044.docx]

**NMR structure elucidation of 6**

The molecular structure of **6** was determined by a combination of 1D and 2D correlation experiments, including ^1^H, ^13^C{^1^H}, ^1^H-^1^H COSY, ^1^H-^1^H NOESY, ^1^H-^13^C HSQC, ^1^H-^13^C HSQC-TOCSY, ^1^H-^13^C HMBC, ^1^H-^15^N HMQC and ^1^H-^15^N HMBC. As several key ^1^H-^13^C and ^1^H-^15^N correlations were not observed due to the broadening of proton signals at 25 °C, ^1^H spectra were measured at variable temperature in order to find more suitable experimental conditions. Consequently, the 1D and 2D spectra were also measured at -40 or -50 °C. Following paragraphs summarize the analysis of 1D and 2D NMR data.

The ^13^C spectrum recorded at 25 °C suggested the presence of three 2-aminoquinolin-4(1*H*)-one units, because there were 27 signals in the range of 175.50-99.85 ppm and these signals formed nine groups. Additional two methylene carbon signals were observed at 35.30 and 18.73 ppm, which were attributed to the links connecting the three units. This conclusion was also supported by the ^1^H spectrum at 25 °C. In addition to 12 C*H* proton signals in the range of 8.3-7.1 ppm suggesting the presence of three 2-aminoquinolin-4(1*H*)-one moieties, four relatively broad signals representing four hydrogen atoms attached to two methylene carbons (35.30 and 18.73 ppm) were observed between 4.9 and 3.7 ppm. In addition to that, there were several relatively narrow signals at 12.98 (1H), 10.81 (1H) and 6.95 ppm (2H), which were tentatively assigned to two NH and one NH_2_ functional groups, respectively. Taking into account the ^1^H and ^13^C chemical shifts of the methylene protons and carbons, we proposed the structure shown in Figure S37. Next, 2D spectra were analyzed in order to assign all ^1^H and ^13^C signals and to confirm the putative structure.

**S37 Fig. Proposed molecular structure of compound 6 and atom numbering used in the NMR structural analysis.**

Figure S38 depicts most important ^1^H-^13^C long-range interactions observed at 25 °C. Most quaternary carbon signals were assigned and the planar structure was almost confirmed. However, we were not able to observe any long-range correlations of the methylene protons H-12 due to the signal broadening present at 25 °C. For this reason, carbon atoms C-2’ and C˗3’ could not be correlated.

**S38 Fig. Key long-range ^1^H-^13^C correlations observed at 25 °C. All correlations of protons H˗7, H-8, H-7’, H-8’, H-7’’ and H-8’’ are omitted for simplicity. Correlations of protons H-6, H-9, H-6’, H-9’, H-6’’ and H-9’’ to protonated-carbons of the same ring are also omitted for clarity.**

The ^1^H-^1^H NOESY spectrum provided several through-space correlations that further supported the proposed structure of **6** and linking among the three structural units as shown in Figure S39.

**S39 Fig. Key ^1^H-^1^H NOE interactions observed in 6 at 25 °C.**

Next, ^1^H-^15^N HMQC and ^1^H-^15^N HMBC spectra were carefully analyzed. Three direct interactions and five long-range (^3^*J*_HN_) interactions were observed, thus leading to detection and assignment of five nitrogen atoms (Figure S40). Only nitrogen H_2_*N*-11’ was not detected.

**S40 Fig. Direct and long-range ^1^H-^15^N correlations observed in 6 at 25 °C.**

Because of the missing long-range correlations of methylene protons H-12 with carbons C-2, C-3, C-4, C-2’, C-3’ and C-4’, and the missing H_2_N-11’ amino group, we measured ^1^H spectra at higher and lower temperature. At -40 °C, the signals of protons H-12 seemed sufficiently narrow and additional signals were observed. For this reason, ^13^C{^1^H} and 2D spectra were collected at this temperature.

Figure S41 shows representative ^1^H-^13^C long-range interactions observed at -40 °C, including correlations of protons H-12. In this case, methylene protons H-12 provided six long-range correlations and enabled us to assign univocally all remaining quaternary carbon signals. The correlations of protons H-12 with carbons C-2, C-3, C-4, C-2’, C-3’ and C-4’ belonging to two neighboring 2-aminoquinolin-4(1*H*)-one units univocally confirmed the putative structure.

**S41 Fig. Key long-range ^1^H-^13^C correlations observed at -40 °C. All correlations of protons H˗7, H-8, H-7’, H-8’, H-7’’ and H-8’’ are omitted for simplicity. Correlations of protons H-6, H-9, H-6’, H-9’, H-6’’ and H-9’’ to protonated-carbons of the same ring are also omitted for clarity.**

Similarly, the ^1^H-^1^H NOESY spectrum recorded at the low temperature provided additional homonuclear through-space correlations. Most importantly, we identified two signals of amino protons N*H*_2_-11’ (Figure S42).

**S42 Fig. Key ^1^H-^1^H NOE interactions observed in 6 at -40 °C.**

Finally, the ^1^H-^15^N HMQC spectra recorded at -40 °C and -50 °C were analyzed. The spectrum recorded at -50 °C yielded six direct ^1^H-^15^N interactions, and hence ^15^N chemical shifts of all nitrogen atoms in the molecule were determined and the broad proton signals were assigned (Figure S43). It is worth of mentioning that the two amino protons H-11’ were not chemically equivalent at -40 °C and -50 °C. This might probably be attributed to a hydrogen-bonding between the amino proton observed at 9.71 ppm and the oxygen atom of carbonyl group at C-4. The presence of hydrogen-bonding between N*H*-11’ and C*O*-4 and also between N*H*-11 and C*O*-4’ relates to the non-equivalency of linking methylene protons by stopping free rotation around bridgehead methylene CH_2_-12 with non-planar conformation. A ^1^H-^15^N HMBC spectrum was recorded at -40 °C, but it did not provide any long-range correlations due to the significant broadening of proton resonances.

**S43 Fig. Direct ^1^H-^15^N correlations observed in 6 at -50 °C.**
